# Identification of an Antiretroviral Small Molecule That Appears To Be a Host-Targeting Inhibitor of HIV-1 Assembly

**DOI:** 10.1128/JVI.00883-20

**Published:** 2021-01-13

**Authors:** Jonathan C. Reed, Dennis Solas, Anatoliy Kitaygorodskyy, Beverly Freeman, Dylan T. B. Ressler, Daryl J. Phuong, J. Victor Swain, Kent Matlack, Clarence R. Hurt, Vishwanath R. Lingappa, Jaisri R. Lingappa

**Affiliations:** a Department of Global Health, University of Washington, Seattle, Washington, USA; b Prosetta Biosciences, San Francisco, California, USA; Icahn School of Medicine at Mount Sinai

**Keywords:** ABCE1, DDX6, HIV-1 assembly, HIV-1 capsid, RNA granules, antiretroviral agent, cell-free system, drug screen, virus-host interactions

## Abstract

Given the projected increase in multidrug-resistant HIV-1, there is an urgent need for development of antiretrovirals that act on virus life cycle stages not targeted by drugs currently in use. Host-targeting compounds are of particular interest because they can offer a high barrier to resistance. Here, we report identification of two related small molecules that inhibit HIV-1 late events, a part of the HIV-1 life cycle for which potent and specific inhibitors are lacking. This chemotype was discovered using cell-free protein synthesis and assembly systems that recapitulate intracellular host-catalyzed viral capsid assembly pathways. These compounds inhibit replication of HIV-1 in human T cell lines and peripheral blood mononuclear cells, and are effective against a primary isolate. They reduce virus production, likely by inhibiting a posttranslational step in HIV-1 Gag assembly. Notably, the compound colocalizes with HIV-1 Gag *in situ*; however, unexpectedly, selection experiments failed to identify compound-specific resistance mutations in *gag* or *pol*, even though known resistance mutations developed upon parallel nelfinavir selection. Thus, we hypothesized that instead of binding to Gag directly, these compounds localize to assembly intermediates, the intracellular multiprotein complexes containing Gag and host factors that form during immature HIV-1 capsid assembly. Indeed, imaging of infected cells shows compound colocalized with two host enzymes found in assembly intermediates, ABCE1 and DDX6, but not two host proteins found in other complexes. While the exact target and mechanism of action of this chemotype remain to be determined, our findings suggest that these compounds represent first-in-class, host-targeting inhibitors of intracellular events in HIV-1 assembly.

**IMPORTANCE** The success of antiretroviral treatment for HIV-1 is at risk of being undermined by the growing problem of drug resistance. Thus, there is a need to identify antiretrovirals that act on viral life cycle stages not targeted by drugs in use, such as the events of HIV-1 Gag assembly. To address this gap, we developed a compound screen that recapitulates the intracellular events of HIV-1 assembly, including virus-host interactions that promote assembly. This effort led to the identification of a new chemotype that inhibits HIV-1 replication at nanomolar concentrations, likely by acting on assembly. This compound colocalized with Gag and two host enzymes that facilitate capsid assembly. However, resistance selection did not result in compound-specific mutations in *gag*, suggesting that the chemotype does not directly target Gag. We hypothesize that this chemotype represents a first-in-class inhibitor of virus production that acts by targeting a virus-host complex important for HIV-1 Gag assembly.

## Abstract (precis)

The success of antiretroviral treatment for HIV-1 is at risk of being undermined by the growing problem of drug resistance. Thus, there is a need to identify antiretrovirals that act on viral life cycle stages not targeted by drugs in use, such as the events of HIV-1 Gag assembly. To address this gap, we developed a compound screen that recapitulates the intracellular events of HIV-1 assembly, including virus-host interactions that promote assembly. This effort led to the identification of a new chemotype that inhibits HIV-1 replication at nanomolar concentrations, likely by acting on assembly. This compound colocalized with Gag and two host enzymes that facilitate capsid assembly. However, resistance selection did not result in compound-specific mutations in *gag*, suggesting that the chemotype does not directly target Gag. We hypothesize that this chemotype represents a first-in-class inhibitor of virus production that acts by targeting a virus-host complex important for HIV-1 Gag assembly.

## INTRODUCTION

In an era in which HIV-1 vaccine trial results have been disappointing, antiretroviral (ARV) drugs stand out as a stunning success story. Treatment with combined antiretroviral therapy (ART) over the past three decades has resulted in enormous reductions in morbidity and mortality from HIV-1 infection, with 62% of the 38 million people living with HIV receiving antiretroviral drugs in 2018 (1). ART is also the mainstay of HIV-1 pre-exposure prophylaxis, a remarkably successful approach to preventing HIV-1 infection (2). In addition, ART will be important for future HIV cure strategies. Unfortunately, the success of HIV ART is at risk of being undermined by the increasingly serious problem of HIV-1 drug resistance (3). Virologic failure is seen in up to 20% of individuals receiving first-line ART in low and middle-income countries (4), with up to half of first-line ART failures in sub-Saharan Africa involving resistance to all three drugs in tenofovir-containing regimens (5). Moreover, the prevalence of drug-resistant HIV-1 is predicted to increase substantially over time as more people receive treatment (6). Indeed, experts have drawn analogies between the future of ART and the current crisis of multidrug-resistant tuberculosis (7). For this reason, identifying novel targets in the HIV-1 life cycle and candidate small molecules that inhibit these targets is a high priority, with the goal of driving development of new ARV drugs.

**FIG 1:**
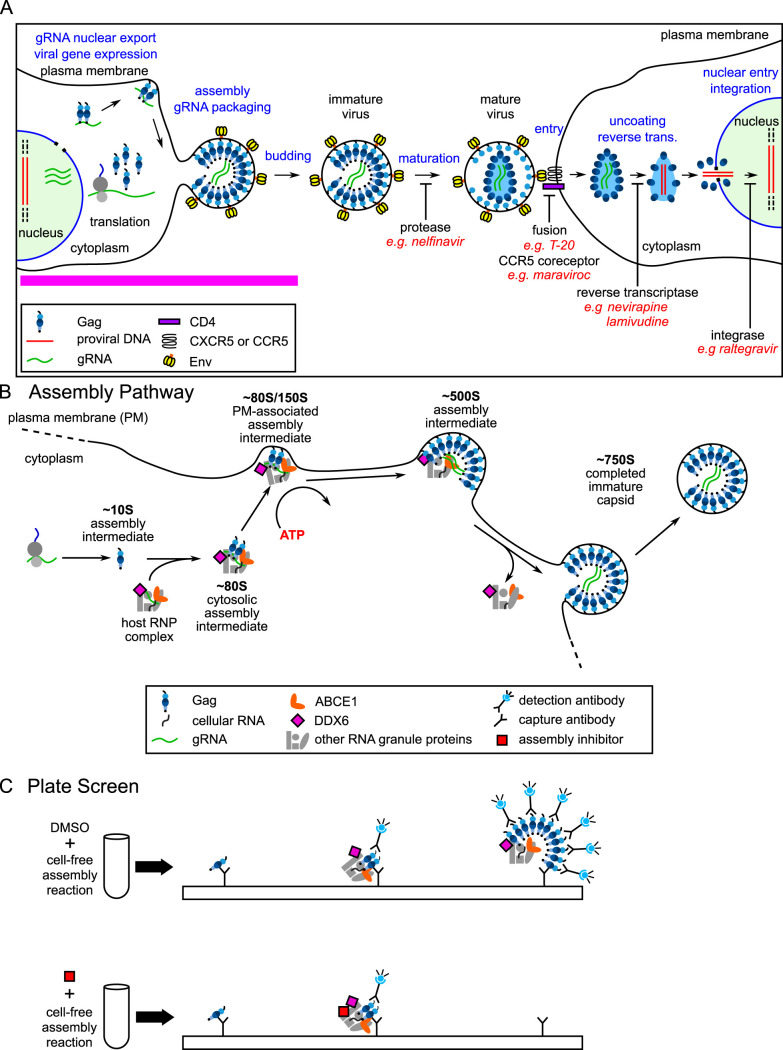
HIV-1 life cycle, assembly pathway, and assembly plate screen. (A) Schematic showing each step of the HIV-1 life cycle, beginning with expression of the integrated provirus, followed by the late and early stages of the life cycle, ending with integration in a newly infected cell. The different stages of the virus life cycle are indicated in blue text. Examples of ARV drugs currently in use are in red text, with black labels under blockade arrows indicating the targets of these drugs. The pink line indicates late events in the viral life cycle that are not targeted by currently approved ARV drugs. (B) Schematic showing the host-catalyzed HIV-1 assembly pathway, starting with Gag synthesis and formation of the early ∼10S assembly intermediate. Next, the cytosolic ∼80S intermediate forms when ∼10S Gag coopts a host RNP complex that contains ABCE1 and the RNA granule protein DDX6, two host enzymes that have been shown to facilitate assembly. The ∼80S assembly intermediate appears to target to the plasma membrane where Gag multimerization continues resulting in formation of the ∼150S and subsequently the ∼500S late assembly intermediate. When assembly of ∼750S immature capsid is completed, the host RNP complex is released. Relevant references are in the text. (C) Schematic showing the cell-free protein synthesis and assembly plate screen that was utilized to identify small molecule inhibitors of the host-catalyzed pathway of HIV-1 assembly. Briefly, anti-Gag antibody (capture antibody) binds Gag monomers, oligomers, and multimers generated in a cell-free assembly reaction. The same Gag antibody is used as a detection antibody that binds to captured oligomers and multimers, but not monomers, in proportion to the amount of multimerization, thereby generating a larger fluorescent signal when multimerization occurs. The upper diagram shows anti-Gag antibodies capturing and detecting Gag oligomers and multimers in assembly intermediates formed during an HIV-1 cell-free assembly reaction carried out in the presence of DMSO, which does not inhibit assembly. The lower diagram shows that adding an assembly inhibitor at the start of the cell-free reaction causes fewer Gag oligomers and multimers to be produced, thereby reducing the detection antibody signal relative to signal in the DMSO control.

Identification of new druggable targets in the poorly understood stages of the viral life cycle will be particularly important for development of drugs aimed at HIV-1 strains resistant to currently available drugs. Mapping drugs currently in use onto the HIV-1 life cycle reveals that many stages of the viral life cycle, including maturation, entry, and postentry events, are targeted by currently available ARV drugs. However, in contrast, there is a striking lack of drugs that target intracellular viral late events (8) (pink bar in Fig. 1A), which include posttranslational steps in immature capsid assembly. Formation of a single infectious virus requires assembly of ∼3,000 copies of the HIV-1 Gag protein, which are synthesized and oligomerize in the cytoplasm and subsequently target to the plasma membrane, where they encapsidate the HIV-1 genome while multimerizing to form the immature capsid. The immature capsid then undergoes budding, release, and maturation (Fig. 1A). Interest in targeting HIV-1 assembly has been high, leading to numerous drug screens in recent years (9, 10). Of the compounds identified in these screens, the most successful have been those that bind to the capsid protein (CA). CA is critical at multiple stages of the viral life cycle, including immature capsid assembly, maturation, infectivity, and postentry events. Interestingly, potent small molecules that target CA do not selectively inhibit immature capsid assembly (reviewed in reference 9); instead, they either act on the mature capsid, targeting virion infectivity and postentry events (e.g., PF74 [11–16]), or they act broadly on Gag synthesis, assembly, and postentry events (e.g., GS-CA1 [17]) (Fig. 1A). Indeed, to our knowledge, there are no reports of any screen identifying a potent small molecule that selectively inhibits intracellular events in HIV-1 assembly.

We hypothesized that one reason for the failure to identify potent and selective inhibitors of HIV-1 assembly might be that previous screens were either very broad or very narrow, i.e., they encompassed the entire viral life cycle (see, for example, references 12 and 18) or they were based solely on multimerization of recombinant CA-derived peptides *in vitro* (see, for example, references 19 to 22). We further hypothesized that a screen that focuses only on events of Gag assembly but includes known cellular facilitators of immature HIV-1 capsid assembly could be more successful than other screens in identifying a potent and selective inhibitor of intracellular events in HIV-1 assembly. Specifically, while recombinant Gag is able to assemble into immature capsid-like particles in the absence of host proteins (reviewed in reference 23), 2 decades of studies support a different model for HIV-1 assembly in cells, one in which Gag assembles into immature capsids via a pathway of assembly intermediates containing viral proteins as well as host proteins that act catalytically to promote HIV-1 capsid assembly (see, for example, references 24 to 34) (Fig. 1B). This model suggests that to succeed in the hostile environment of the cytoplasm, Gag may have evolved to utilize host proteins to catalyze Gag multimerization, promote RNA packaging, and sequester assembly within host complexes where nascent virions would be less vulnerable to host defenses. If this host-catalyzed model of HIV-1 capsid assembly in the cytoplasm is valid, then a screen that recapitulates this pathway might succeed in identifying new druggable targets and novel antiretroviral small molecules.

Indeed, a precedent exists for a screen that recapitulates a host-catalyzed assembly pathway enabling identification of a novel antiviral target and small molecule inhibitor. Previously our group, in collaboration with investigators at the Centers for Disease Control and Prevention, used a cell-extract-based screen that recapitulated an intracellular assembly pathway for rabies virus (RABV) to identify the first reported small molecule inhibitor of RABV replication in cell culture (35). Notably, this small molecule binds to a multiprotein complex that contains ATP-binding cassette protein E1 (ABCE1), a host enzyme we had previously identified in HIV-1 assembly intermediates, suggesting that similar host complexes may be involved in the assembly of diverse viruses.

Given the success of the cell-free screen for identifying inhibitors of RABV assembly, we reasoned that a similar cell-free assembly pathway screen could be used to identify novel inhibitors of HIV-1 assembly. Indeed, the HIV-1 immature capsid assembly pathway that we sought to inhibit was originally identified in a cell-free system (28). Adapted from the *in vitro* protein synthesis systems that were used to identify signal sequences (36), the cell-free HIV-1 assembly system supports *de novo* synthesis of HIV-1 Gag polypeptides from a Gag mRNA using energy substrates, amino acids, and a cellular extract that provides host factors required for Gag translation and posttranslational events of Gag assembly. When programmed with wild-type Gag mRNA, this system produces particles that closely resemble completed immature HIV-1 capsids generated by provirus-expressing cells, judging by their ultrastructural appearance and their size and shape (as defined by a sedimentation value of ∼750S [28]). Two complementary approaches initially suggested that immature HIV-1 capsid assembly progresses through a pathway of assembly intermediates: first, pulse-chase studies in the cell-free system revealed sequential progression of HIV-1 Gag through complexes of increasing size (∼10S to ∼80S/150S to ∼500S to ∼750S), consistent with these complexes being intermediates in a pathway that culminated in the formation of the ∼750S completely assembled immature capsid. Second, Gag mutants defined by others to be assembly-defective in cells were arrested at specific steps of the cell-free assembly pathway, while assembly-competent Gag mutants progressed through the entire pathway (28, 37). Notably, biochemical analysis demonstrated that posttranslational events in this assembly pathway required ATP, indicating that HIV-1 immature capsid assembly in cells is energy dependent (28) (Fig. 1B).

While initially identified in a cell-free system, the HIV-1 capsid assembly pathway has been largely studied in cellular systems in the last 2 decades. Key features of the assembly pathway were validated in cells expressing the HIV-1 provirus (reviewed in reference 32), including the sequential progression of Gag through the pathway of assembly intermediates (26, 32), the energy dependence of the pathway (25), and the arrest of known assembly-defective Gag mutants at specific steps in the pathway (25–28, 32, 33, 38). The energy dependence of immature capsid assembly, which has been confirmed by other groups (39), was subsequently explained by the finding that the assembly intermediates contain at least two host enzymes that facilitate assembly: the ATPase ABCE1 and the DEAD box RNA helicase 6 (DDX6) (30, 34). Other studies suggest that packaging of the HIV-1 genome appears to occur in the assembly intermediates (24, 32) and that other lentiviruses utilize analogous assembly pathways (25, 31). Immunoprecipitation and imaging studies confirmed the association of ABCE1 and DDX6 with assembling Gag and identified two other host enzymes in assembly intermediates, AGO2 and DCP2 (24, 26, 27, 30–33). The finding that assembly intermediates contain proteins typically found in host RNA granules, e.g., DDX6, AGO2, and DCP2, supports a model in which assembling Gag coopts a unique subclass of host ribonucleoprotein (RNP) complexes that are related to RNA granules but differ from well-studied RNA granules such as P-bodies and stress granules. RNA granules are a diverse group of RNP complexes involved in all aspects of host RNA metabolism except protein synthesis. Sequestration of immature HIV-1 capsid assembly events in such host RNP complexes could allow HIV-1 assembly to be protected from the innate immune system while at the same time allowing assembling Gag access to the viral genome, which is found in these complexes (24, 32), and to utilize enzymes that could facilitate packaging and assembly (Fig. 1B).

Motivated by our success in using a RABV cell-free screen to identify a small molecule that potently inhibits RABV in cell culture, we sought to identify a small molecule inhibitor of the HIV-1 assembly pathway through the use of cell-free assembly screens. Here, we report our identification of a potent antiretroviral chemotype that inhibits HIV-1 replication, represented by the small molecule PAV-117 and its more potent analog PAV-206. Our studies suggested that this chemotype inhibits HIV-1 replication by interfering with HIV-1 assembly. Moreover, we found that a biotinylated analog of these compounds colocalizes with Gag *in situ*, raising the possibility that the compounds target HIV-1 Gag. However, surprisingly, compound-specific mutations did not arise in *gag* or *pol* after 37 weeks of PAV-206 selection in HIV-1-infected human cells, in stark contrast to nelfinavir selection examined in parallel, arguing against Gag being the direct target of PAV-206. Additional imaging studies shed more light on our failure to identify viral resistance mutations by demonstrating that the biotinylated PAV-206 analog also colocalizes with two host proteins associated with Gag in intracellular HIV-1 capsid assembly intermediates, the enzymes ABCE1 and DDX6. Notably, the biotinylated PAV-206 analog failed to colocalize with two other host proteins, the Ras-GAP SH3 domain-binding protein 1 (G3BP1) and 5′–3′ exoribonuclease 1 (XRN1), which are found in stress granules and P-bodies, respectively. G3BP1 and XRN1 were examined because they have not been found associated with HIV-1 Gag in HIV-1-expressing cells, to our knowledge. While the exact target and mechanism of action of PAV-117/PAV-206 remain to be determined, these studies suggest that these compounds represent a class of novel host-targeting ARV small molecules that inhibits HIV-1 virus production by selectively acting on host-protein-containing capsid assembly intermediates.

## RESULTS

### Development of a novel screen for HIV-1 assembly inhibitors based on previous studies of the intracellular HIV-1 capsid assembly pathway.

Given that the studies described above had confirmed that HIV-1 assembly intermediates are present in HIV-1-expressing cells, we reasoned that the cell-free system in which these intermediates were first identified could hold promise as a drug screen. Just as the cell-free system allowed detection of intracellular events of assembly that were present but harder to detect in living cells, so also a cell-free drug screen might reveal small molecules targeting those hard-to-detect steps. Such a cell-free screen for HIV-1 assembly inhibitors would have the advantage of recapitulating evolutionarily conserved virus-host interactions critical for immature HIV-1 capsid assembly while being much less expensive and less laborious than drug screens that utilize infected cell lines in culture. In addition, the cell-free system can be adapted for a moderately high-throughput format.

For these reasons, we adapted the HIV-1 cell-free assembly reaction to generate a drug screen (Fig. 1C) similar to the RABV drug screen (35). Cell-free reactions, upon completion, contain assembly intermediates and fully assembled immature capsid-like particles, unless performed in the presence of an inhibitor of assembly, in which case Gag will be largely unassembled (i.e., monomers and dimers). Plate-bound anti-Gag antibodies would be expected to capture monomeric, dimeric, and multimerized Gag from the reaction. To determine how much Gag multimerization occurred during the reaction, a soluble anti-Gag detection antibody is added. The detection antibody does not bind Gag monomers (since the antigenic site on the monomer is already bound by capture antibody) but would be expected to bind to unoccupied Gag proteins in multimers. Signal from bound detection antibody is quantified using a fluorescent detection system, with more signal being generated by Gag multimers. Thus, an effective inhibitor of Gag multimerization will be recognized by a reduction in detection antibody signal (Fig. 1C). Because we are interested in inhibitors of assembly rather than protein synthesis, we utilized a counter screen to eliminate from further consideration all small molecules that inhibit translation of green fluorescent protein (GFP) in cell-free reactions.

### Identification of a potent inhibitor of HIV-1 replication.

**FIG 2:**
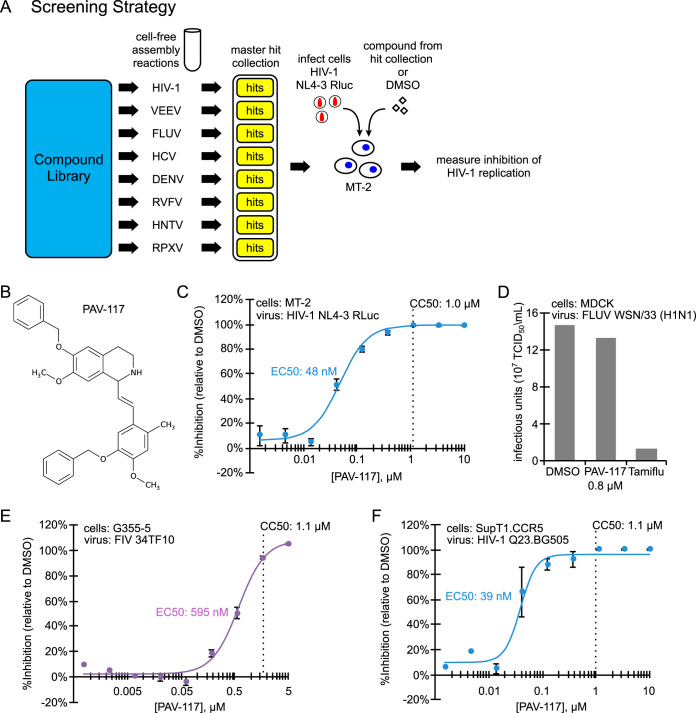
PAV-117, identified using cell-extract-based assembly screens, inhibits replication of HIV-1, but not FIV or FLUV, in cell culture. (A) Schematic showing the cell-free assembly screening strategy used to identify PAV-117. Small molecules from a compound library of 150,000 compounds were assayed in cell-free screens that recapitulate assembly of eight different viral capsids, including HIV-1, Venezuelan equine encephalitis virus (VEEV), influenza A virus (FLUV), hepatitis C virus (HCV), dengue virus (DENV), Rift Valley fever virus (RVFV), Hantaan virus (HNTV), and rabbit pox virus (RPXV). Each screen is analogous to the HIV-1 screen (see Fig. 1C). Results from the library screens led to generation of a master hit collection of 249 small molecules that displayed inhibitory activity in one or more of the eight cell-free assembly screens. Compounds in the master hit collection were assayed for inhibition of HIV-1 replication in MT-2 T cells, with PAV-117 identified as the compound with optimal EC_50_, CC_50_, and other characteristics in the MT-2 assay. (B) Chemical structure of PAV-117, a tetrahydroisoquinolone. (C) To determine the EC_50_ of PAV-117 against HIV-1 replication, a dose-response curve for inhibition of HIV-1 replication by PAV-117 was generated by treating human MT-2 T cells with the indicated doses of PAV-117, followed by infection with a replication-competent HIV-1 NL4-3 RLuc reporter virus (MOI of 0.02). After 96 h of spreading infection, luciferase activity was measured as an indicator of HIV-1 replication and is displayed as the inhibition of replication relative to DMSO-treated controls (% inhibition). The CC_50_ was determined in parallel using uninfected MT-2 T cells and is marked by a vertical dashed line. Error bars show the SEM determined from three replicates. (D) Graph showing quantification of infectious FLUV in MDCK cells treated with DMSO or with either PAV-117 or Tamiflu at a concentration 20-fold higher than the EC_50_ for each drug (0.8 μM for PAV-117, 10 μM for Tamiflu). Treated cells were infected with FLUV (strain WSN/33 [H1N1], MOI of 0.001), and the viral titers were measured after 24 h by TCID_50_ and are shown as infectious units (10^7^ TCID_50_ U/ml). (E) To determine the EC_50_ of PAV-117 against FIV replication, a dose-response curve for the inhibition of FIV replication by PAV-117 was generated by treatment of feline G355-5 cells with the indicated doses of PAV-117, followed by infection with FIV 34TF10 (1,954 nU of RT activity per well). After 144 h of spreading infection, RT activity was measured and used to calculate inhibition of FIV replication relative to DMSO controls (% inhibition). The CC_50_ was determined in parallel using uninfected G355-5 cells and is marked by a vertical dashed line. Error bars show the SEM determined from three replicates. (F) Quantification of replication of an HIV-1 primary isolate. To determine the EC_50_ of PAV-117 against HIV-1 replication, a dose-response curve for inhibition of HIV-1 replication by PAV-117 was generated by treating human SupT1.CCR5 cells with the indicated doses of PAV-117 or DMSO, followed by infection with HIV-1 Q23.BG505, a CCR5-tropic subtype A molecular clone. HIV-1 replication proceeded for 96 h and was followed by measurement of HIV-1 infectivity in the culture supernatant using the MUG assay in TZM-bl cells.

Once this cell-free HIV-1 assembly screen and counterscreen were successfully established and validated, similar cell-free screens were established for quantifying assembly of the capsids of seven other viruses (Fig. 2A). A compound library of ∼150,000 small molecules with drug-like characteristics was screened against these eight cell-free assembly assays, resulting in a master hit collection of 249 small molecules that inhibited assembly in one or more virus assembly screen(s) but did not significantly inhibit GFP translation.

Compounds in the master hit collection were further screened for their ability to inhibit replication of HIV-1 in the MT-2 human T cell line. The compound with the most favorable characteristics in this assay was PAV-117, a tetrahydroisoquinolone derivative with excellent drug-like properties (Fig. 2B). PAV-117 inhibits replication of HIV-1 NL4-3 in MT-2 cells with a 50% reduction in virus replication (EC_50_) of 48 nM and a 50% reduction in cell viability (CC_50_) of 1.0 μM (Fig. 2C). The nanomolar EC_50_ and a selectivity index (CC_50_/EC_50_) of 21 make PAV-117 an excellent compound for further optimization. Notably, PAV-117 was not active against influenza A virus WSN/33 (H1N1) (FLUV) suggesting that it is not an antiviral that acts broadly on all enveloped viruses (Fig. 2D). Moreover, PAV-117 was also relatively inactive against feline immunodeficiency virus (FIV), a nonprimate lentivirus that is related to HIV-1, with the EC_50_ (595 nM) for inhibition of FIV replication in feline G355-5 cells being <2-fold lower than the CC_50_ in G355-5 cells (1.1 μM; Fig. 2E). Thus, PAV117 displays specificity even among lentiviruses. Importantly, PAV-117 was as active against a primary isolate of HIV-1 as against an HIV-1 lab isolate (the primary isolate EC_50_ of 39 nM in Fig. 2F versus the lab isolate EC_50_ of 48 nM in Fig. 2C), indicating that its antiretroviral activity is not restricted to laboratory isolates of HIV-1.

### Defining the step in the viral life cycle at which the novel small molecule acts.

**FIG 3:**
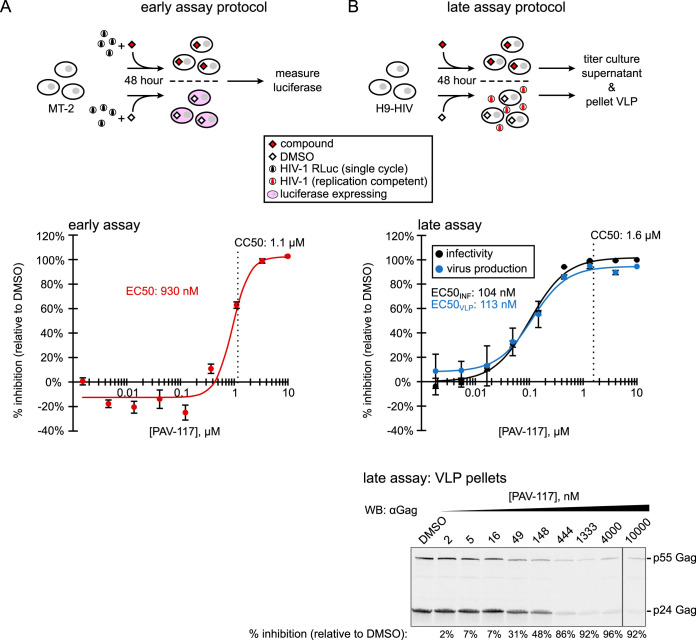
PAV-117 acts late in the HIV-1 life cycle, inhibiting virus production but not specific infectivity. (A) Schematic of the early assay, which measures effects on viral entry through early viral gene expression. MT-2 cells were infected with the single-round HIV-1 pNL4-3 RLuc virus (*env*-deleted and pseudotyped with HIV-1 NL4-3 Env) in the presence of compound or DMSO. After 48 h, luciferase activity was measured and used to calculate inhibition of HIV-1 infection relative to the DMSO control (% inhibition). The graph shows the dose-response curve for inhibition of HIV-1 early events by PAV-117 that was generated using this assay and used to determine the EC_50_. The CC_50_ was determined in parallel using uninfected MT-2 T cells and is marked by a vertical dashed line. Error bars in the graph show the SEM determined from three replicates. (B) Schematic of the late assays, which measure the effects on viral late events, starting with the expression of Gag and GagPol through virus release and maturation. Chronically infected H9 T cells (H9-HIV) were treated with either compound or DMSO, and media collected 48 h later were used for two assays: (i) to quantify inhibition of HIV-1 infectivity relative to DMSO control by titering on TZM-bl cells (black curve, used to calculate the EC_50_ for the inhibition of infectivity) and (ii) to quantify inhibition of virus production by pelleting virus for Western blot (WB) with antibody to HIV-1 Gag (αGag; blue curve, used to calculate the EC_50_ for the inhibition of virus production). The CC_50_ was determined in the inhibition assay and is marked by a vertical dashed line. A representative αGag WB of virus pellets is shown below the dose-response graph, with DMSO treatment or concentration of PAV-117 indicated above each WB lane and the percent inhibition of virus production (relative to the DMSO-treated control) indicated below each lane. The error bars in panels A and B show the SEM determined from two replicates.

Since other types of assembly inhibitor screens had identified compounds that act on viral early events as described above, we next sought to determine whether PAV-117 inhibits HIV-1 replication by acting on entry or postentry events in the HIV-1 life cycle. For this assay, we infected MT-2 cells, in the presence of PAV-117 or dimethyl sulfoxide (DMSO), with a luciferase-encoding HIV-1 reporter virus (HIV-1 pNL4-3 RLuc Δ*env*) that is pseudotyped with HIV-1 Env and undergoes only one round of replication (Fig. 3A, diagram). At nontoxic concentrations, PAV-117 did not reduce luciferase activity in this assay (Fig. 3A, graph; EC_50_ of 930 nM; CC_50_ of 1.1 μM), indicating that it does not act on early events in the HIV-1 life cycle. The effect of PAV-117 on viral late events (events that occur after integration) was assayed using chronically infected H9 T cells, which produce infectious virus from integrated HIV-1 provirus (Fig. 3B, diagram). In this assay, PAV-117 inhibited production of infectious virus in the culture supernatant, with an EC_50_ of 104 nM (Fig. 3B, black dose-response curve in graph). Thus, PAV-117 activity can be entirely attributed to inhibition of viral late events. To determine whether PAV-117 results in production of virus-like particles (VLP) that were noninfectious, we quantified production of p24 Gag in VLP pelleted from cell culture supernatants of chronically infected H9 cells treated with PAV-117 or DMSO. Finding that PAV-117 reduces virus infectivity without altering the amount of p24 Gag in virus pellets would indicate that PAV-117 results in production of noninfectious VLP. However, what we actually observed was the opposite—that inhibition of p24 production was similar to inhibition of supernatant infectivity (EC_50_s of 113 nM versus 104 nM, respectively; compare blue versus black curve in Fig. 3B, graph), indicating that the compound blocks virus production. Western blots of VLP in supernatants also showed no change in the ratio of p55 to p24 with increasing PAV-117 concentration relative to DMSO treatment (Fig. 3B, blot), arguing that it does not act on Gag cleavage required for virus maturation.

**FIG 4:**
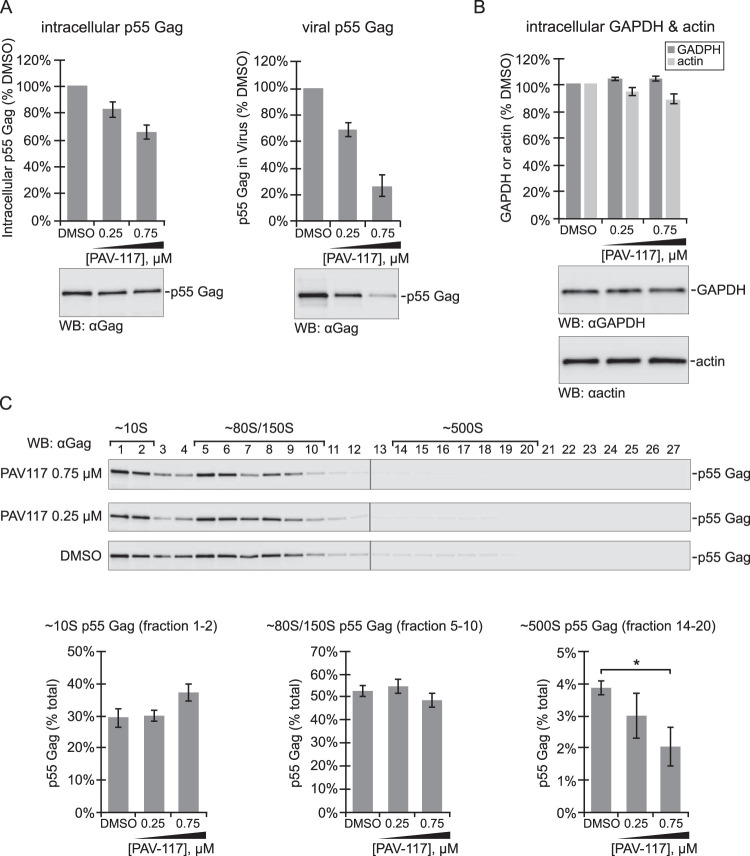
PAV-117 appears to act during the HIV-1 assembly pathway, after formation of the ∼80S/150S intermediate. MT-2 cells were infected with HIV-1 LAI *pro*– Δ*env* (pseudotyped with HIV-1 NL4-3 Env) and treated with DMSO or the indicated concentrations of PAV-117 for 48 h. (A) Cell lysates and media were harvested to analyze effects on intracellular steady-state p55 Gag levels and p55 Gag in VLP, as indicated, using WB with Gag antibody (αGag) to quantify p55 Gag (no p24 was produced due to the use of a protease-deficient virus for infection). (B) Cell lysates were also analyzed for intracellular steady-state levels of two cellular proteins, GAPDH and actin, by WB with αGAPDH and αactin. For panels A and B, the data in the graphs are shown as the percentage of DMSO-treated controls, with error bars showing the SEM from three replicates, and representative WBs are shown below graphs. (C) To quantify intracellular steady-state levels of assembly intermediates, cell lysates from panels A and B were also analyzed by velocity sedimentation, followed by WB of each gradient fraction with αGag. Fraction numbers are indicated above the WB panels, with migration of specific assembly intermediates indicated by brackets above. Graphs show quantification of p55 Gag in fractions containing the ∼10S, ∼80S/150S, and ∼500S intermediates as percentages of the total p55 Gag in the gradient. The expected migration of each protein in WB panels is indicated on the right. Error bars show the SEM determined from three replicates. The ∼500S intermediate is the only intermediate for which a significant difference is observed between the DMSO and 0.75 μM PAV-117 groups, as indicated by an asterisk (*P* < 0.05).

The finding that the entire effect of PAV-117 is due to inhibition of virus production (Fig. 3) indicated that PAV-117 likely acts on an intracellular viral late event. Such events include transcription of the full-length HIV-1 mRNA, translation of this mRNA to produce Gag and GagPol, assembly of Gag to produce immature capsids, and budding at the plasma membrane to release immature virus. We hypothesized that PAV-117 likely acts on posttranslational events in assembly of immature HIV-1 capsids for two reasons: first, because a counter screen had been utilized to eliminate inhibitors of translation, and second, because the cell-free screens that identified PAV-117 recapitulate capsid assembly but not virus budding, release, or maturation. To test this hypothesis, we analyzed MT-2 cells that were infected with a protease-deficient provirus and were treated for 48 h with DMSO or PAV-117 at 0.25 nM versus 0.75 nM (∼EC_70_ and ∼EC_90_). Protease-deficient provirus was used for infections so that only p55 Gag would be present in all samples, thereby allowing direct comparisons of Gag in intracellular and VLP samples, as well as in different assembly intermediates. As expected, we observed a dose-dependent reduction in VLP release into the supernatant (Fig. 4A). Consistent with PAV-117 not being a global inhibitor of protein synthesis, steady-state levels of actin and GAPDH were minimally affected (Fig. 4B). At the highest PAV-117 concentration tested, some reduction in steady-state intracellular p55 Gag levels was observed (Fig. 4A), albeit less than the reduction in VLP production. To further define where PAV-117 acts, we also examined steady-state levels of HIV-1 capsid assembly intermediates. The distinct S values of these intermediates allow them to be separated by velocity sedimentation; together, they represent all the different pools of intracellular Gag found at steady state. PAV-117 resulted in a in a dose-dependent decrease in the steady-state level of the ∼500S intermediate as a percentage of total Gag but did not reduce levels of other assembly intermediates as a percentage of total Gag (Fig. 4C). While the steady-state data in Fig. 4 provide a snapshot of intracellular Gag levels at only one point in time, taken together with the reduction in cumulative VLP production (across 48 h) in Fig. 3A, these data suggest that PAV-117 acts during virus assembly in a manner that reduces steady-state levels of the ∼500S assembly intermediate.

### Generation of analogs of the novel small molecule inhibitor that are more potent or contain a tag.

**FIG 5:**
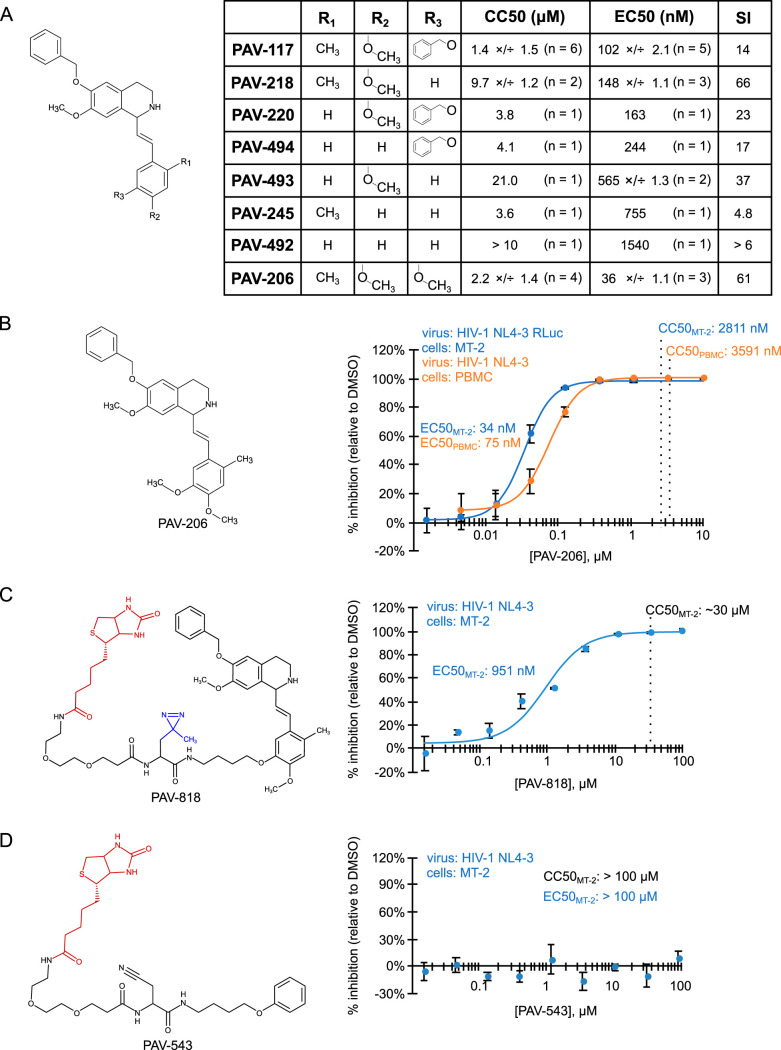
Analysis of structure-activity relationships identified an analog that potently inhibits HIV-1 replication in PBMCs and a site for tags. (A) The general chemical structure of PAV-117 analogs is shown on the left, indicating the R1, R2, and R3 positions in the pendant benzene ring. The table shows results obtained for analogs in which the R1, R2, and R3 positions contain hydrogen, methyl, methoxy, or benzoyloxy groups as indicated, including the EC_50_ for inhibition of HIV-1 replication in MT-2 cells and the CC_50_ in MT-2 cells (assays described in Fig. 2C). Values are shown as the average of multiple independent repeats ×/÷ the GSD, with *n* = the number of independent repeats. Also shown is the selectivity index (SI), which is equivalent to CC_50_/EC_50_. (B) The structure of PAV-206 is shown on the left. The blue dose-response curve shows the inhibition of HIV-1 replication by PAV-206 in MT-2 T cells (using the assay described in Fig. 2C). The orange dose-response curve shows inhibition of HIV-1 replication by PAV-206 in PHA-activated PBMCs infected with unmodified HIV-1 NL4-3 at an MOI of 0.008. (C) On the left is the structure of PAV-818, the biotinylated analog of PAV-206, with the biotin moiety shown in red. Shown on the right is a dose-response curve for inhibition of HIV-1 replication by PAV-818 in MT-2 T cells (assay as in Fig. 2C). In the PAV-818 structure, a diazirine group is shown in blue. This group was added for future cross-linking studies but is not used in the present study. (D) Shown on the left is the structure of PAV-543, a biotinylated compound that does not have antiretroviral activity, with the biotin moiety in red. Shown on the right is a dose-response curve for inhibition of HIV-1 replication by PAV-543 in MT-2 T cells (assay as in Fig. 2C). For all graphs, the indicated EC_50_ values were determined from the dose-response curves. CC_50_ values were determined in uninfected cells in parallel and are marked by vertical dashed lines. Error bars in graphs show the SEM from three replicates.

If PAV-117 acts during assembly, one might expect it to be associated with assembling HIV-1 Gag. To test whether this is the case, we needed an analog of PAV-117 that contains a tag, such as biotin, to allow detection while retaining antiviral activity. To identify positions that could be used for a biotin tag, structure-activity relationships were analyzed with combinations of methyl, methoxy, or benzoyloxy substitutions in the R1, R2, and R3 positions of the pendant benzene ring (Fig. 5A). Interestingly, we observed that maintaining a methyl in the R1 position and a methoxy in the R2 position while altering the R3 position led to compounds that had reduced toxicity (higher CC_50_) and a considerably higher selectivity index when analyzed in HIV-1-infected MT-2 T cells (PAV-218 and PAV-206 in Fig. 5A). PAV-206, which differs from PAV-117 only because it contains a methoxy rather than a benzoyloxy group in the R3 position, was particularly notable since it had a lower EC_50_ and higher CC_50_ than PAV-117, resulting in excellent average selectivity index values of 61 in MT-2 cells (Fig. 5A and B) and 48 in HIV-1-infected peripheral blood mononuclear cells (PBMCs) (Fig. 5B).

In addition to identifying PAV-206, a more potent analog of PAV-117, the structure-activity relationship analysis led to a strategy for introducing a tag into PAV-117 (or PAV-206) by revealing that the R3 position can tolerate either a bulky group (as in PAV-117) or a hydrogen (as in PAV-218) without a significant loss of activity (Fig. 5A). For this reason, we introduced a biotin at this position, generating the PAV-818 analog (Fig. 5C), which is identical to PAV-117 and PAV-206 except at the R3 position. PAV-818 has a higher EC_50_ (951 nM) than the PAV-117 or PAV-206 analogs, but it also has a higher CC_50_ (∼30 μM), generating a selectivity index of ∼32 (Fig. 5C), which is slightly better than the selectivity index of PAV-117. Thus, PAV-818 represents a biotinylated analog of PAV-206 and PAV-117 that can be utilized to visualize where these compounds localize within infected cells.

### The biotinylated analog of PAV-206 colocalizes with HIV-1 Gag *in situ* in infected cells.

**FIG 6:**
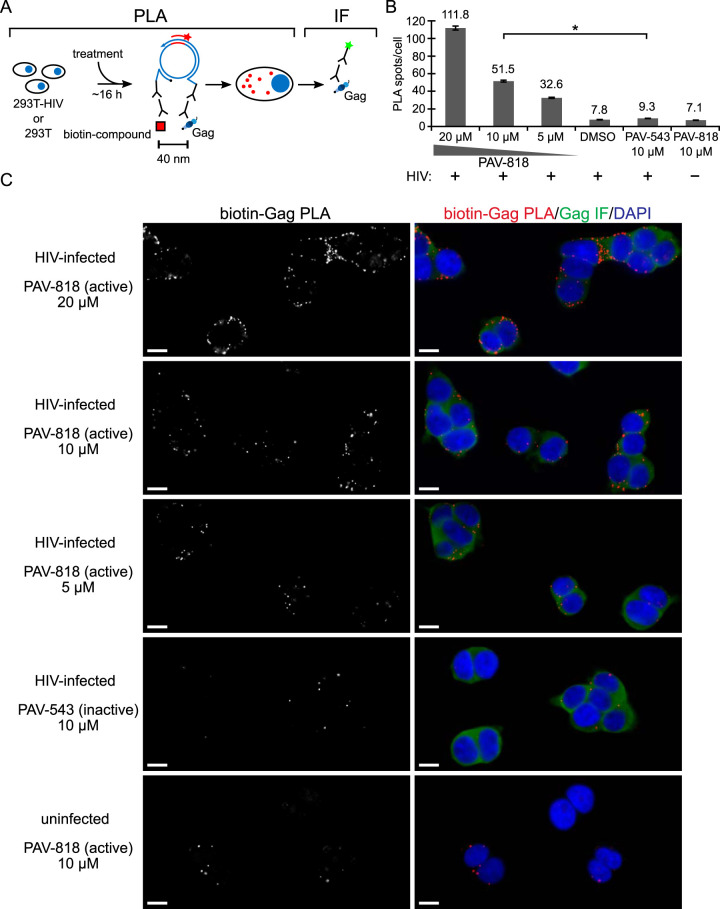
The biotinylated antiretroviral analog of PAV-206 colocalizes with HIV-1 Gag *in situ*. (A) Schematic of the PLA approach for detecting colocalization of compound with Gag. 293T cells chronically infected with HIV-1 (293T-HIV) or uninfected 293T cells were treated with indicated amounts of PAV-818 (the biotinylated active compound), PAV-543 (the biotinylated inactive compound), or DMSO for 16 h. PLA was performed by incubating with primary antibodies (rabbit anti-biotin and mouse anti-Gag), followed by PLA secondary antibodies (anti-rabbit IgG coupled to [+] PLA oligonucleotide and anti-mouse IgG coupled to [–] PLA oligonucleotide). The addition of other PLA reagents leads to connector oligonucleotides linking the “+” and “–” oligonucleotides only if the primary antibodies are colocalized; this in turn results in the PLA amplification reaction. The addition of an oligonucleotide that recognizes a sequence in the amplified regions and is coupled to a red fluorophore (red star) results in intense spots at sites where biotinylated compound and Gag are colocalized *in situ*. After PLA, IF was performed by adding secondary antibody conjugated to a green fluorophore (green star) to detect any unoccupied Gag antibody, thus marking Gag-expressing cells with low-level green fluorescence. (B) Graph showing the average number of biotin-Gag PLA spots per cell for each condition, with “+” indicating HIV-1-infected cells and “–” indicating uninfected cells. Twenty fields were analyzed for each group (containing a total of 186 to 316 cells per group), with error bars showing SEM. *, Significant difference in the number of biotin-Gag PLA spots per cell when comparing treatment with PAV-818 versus PAV-543, both at 10 μM (*P* < 0.001). (C) A representative field for each group quantified in panel B is shown, except for DMSO treatment. Fields on the left show biotin-Gag PLA spots alone in grayscale. To the right are the same fields shown as a merge of three color channels: biotin-Gag PLA (red), Gag IF (green), and DAPI-stained nuclei (blue). Scale bars, 10 μm.

**FIG 7:**
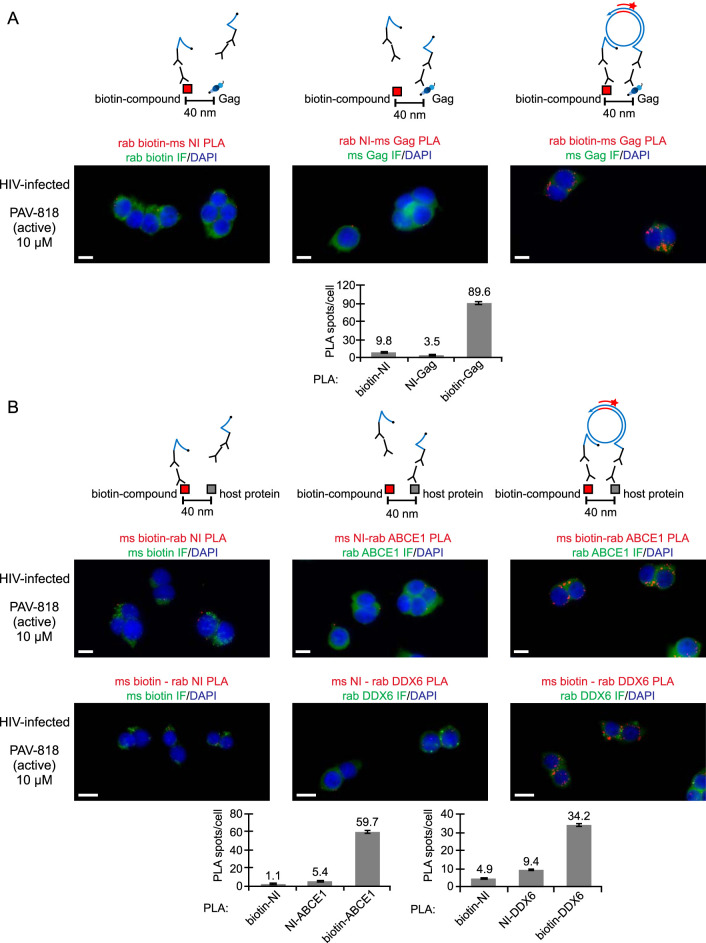
Nonimmune controls for the biotin-Gag, biotin-ABCE1, and biotin-DDX6 proximity ligation assays. (A) NI controls for the biotin-Gag PLA. Above each PLA image is shown the schematic corresponding to the PLA approach in that panel. 293T cells chronically infected with HIV-1 (293T-HIV) were treated with 10 μM PAV-818 (the biotinylated active compound) for 16 h. For the positive control (image and schematic on the right), PLA was performed by incubation with primary antibodies, mouse anti-Gag and rabbit anti-biotin, followed by PLA secondary antibodies and other reagents as described in Fig. 6. In the two negative controls (image and schematic at left and center), one primary antibody was replaced with a NI control antibody from the same species, as indicated. Red spots indicating colocalization of the biotinylated compound with Gag should be absent when either primary antibody is replaced by a NI antibody. After PLA, IF was performed to mark either Gag-expressing or biotin-containing cells with low-level green fluorescence. Images show a representative field for each of the three antibody pairs. Fields are shown as a merge of three color channels: the red channel shows biotin-NI PLA, NI-Gag PLA, or biotin-Gag PLA, as indicated by red labeling above images; the green channel shows biotin IF or Gag IF, as indicated by green labeling above images; and the blue channel shows DAPI-stained nuclei. Scale bars, 10 μm. Graph below shows the average number of PLA spots per cell for each antibody pair. A total of 10 to 20 fields were analyzed for each group (containing 118 to 213 cells per group), with error bars showing the SEM. (B) NI controls for the biotin-ABCE1 PLA (top row of images) and biotin-DDX6 PLA (bottom row of images). Figure organization and 293T-HIV treatments are as in panel A above. For the positive control (images and schematic on the right), PLA was performed by incubation with primary antibodies, rabbit anti-host protein (ABCE1 in top row; DDX6 in bottom row) and mouse anti-biotin, followed by PLA secondary antibodies and other reagents as described in Fig. 6. In the two negative controls (images and schematics at left and center), one primary antibody was replaced with a NI control antibody from the same species, as indicated. Red spots indicating colocalization of the biotinylated compound with the host proteins ABCE1 and DDX6 should be absent when either primary antibody is replaced by a NI antibody. After PLA, IF was performed with the indicated antibody (green fluorescence). Images show a representative field for each of the three antibody pairs. Fields are shown as a merge of three color channels: the red channel shows biotin-NI PLA, NI-host protein PLA, or biotin-host protein PLA, as indicated by red labeling above images; the green channel shows biotin IF or host protein IF, as indicated by green labeling above images; and the blue channel shows DAPI-stained nuclei. Scale bars, 10 μm. Graphs below shows the average number of PLA spots per cell for each antibody pair (ABCE1 on the left; DDX6 on the right). Five fields were analyzed for each group (containing a total of 43 to 77 cells per group), with error bars showing the SEM.

Next, we utilized the biotinylated antiviral analog PAV-818 to ask if this family of compounds colocalizes with HIV-1 Gag as one would expect if they inhibit assembly, as suggested by data in Fig. 3 and 4. For this purpose, we used the proximity ligation assay (PLA), a technique that marks two antigens that are in close proximity with fluorescent spots. Briefly, in PLA, primary antibodies from two species are detected by species-specific secondary antibodies that are tagged with oligonucleotides. These oligonucleotides hybridize to connector oligonucleotides only if the antigens detected by the primary antibodies are within ∼40 nm of each other. Ligation of connector oligonucleotides and the addition of a polymerase results in rolling circle amplification to generate a sequence that can be detected by a complementary oligonucleotide conjugated to a fluorescent probe (40). Thus, the fluorescent spots (in this case, red spots) represent sites where two antigens are colocalized. To determine whether PAV-818 colocalizes with HIV-1 Gag, we performed biotin-Gag PLA on 293T cells that were chronically infected with HIV-1 and treated with either the biotinylated antiretroviral PAV-818, a biotinylated compound that lacks antiviral activity termed PAV-543 (Fig. 5D), or the DMSO vehicle. Quantification of red fluorescent spots, which represent sites where biotinylated compound and Gag are colocalized, revealed 5.5-fold more spots in cells treated with 10 μM PAV-818 than in cells treated with an equivalent concentration of the control compound PAV-543, and a slightly greater difference relative to DMSO-treated cells (Fig. 6A to C). Moreover, the number of biotin-Gag spots observed with PAV-818 treatment displayed a dose-response relationship (Fig. 6B and C). Controls revealed that only a background level of spots was observed when uninfected 293T cells were treated with PAV-818, as would be expected given that uninfected cells lack Gag (Fig. 6B and C). In addition, as expected, when antibody to biotin was replaced with a nonimmune (NI) control antibody only a few spots were observed; similar results were observed when antibody to Gag was replaced with a NI control (Fig. 7A). Together, these data provide evidence that PAV-818 colocalizes with Gag in HIV-1-infected human cells.

### Compound-specific resistance mutations were not observed in *gag* or *pol* after 37 weeks of selection with PAV-206.

**FIG 8:**
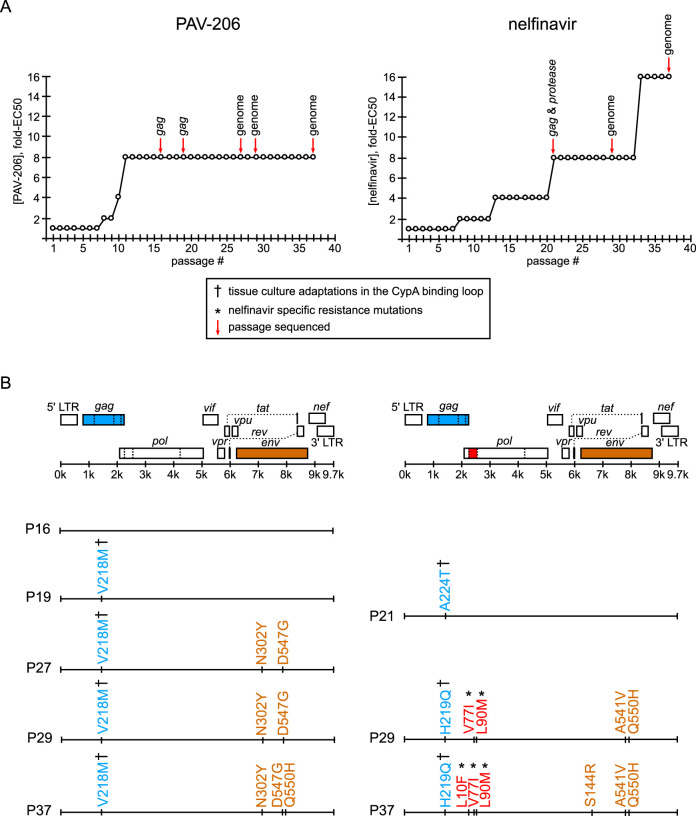
Resistance mutations in *gag* or *pol* were not observed upon selection with PAV-206 in cell culture. (A) An NL4-3 virus stock was passaged weekly for 37 weeks in the presence of either PAV-206 or nelfinavir starting at a concentration of 1× EC_50_. The concentration of compound was increased 2-fold when compound-treated cultures reached maximum viral CPE in a time frame similar to a parallel DMSO control. The graph shows the compound concentration as the fold-EC_50_ versus week of selection (passage number) for PAV-206 (left) or nelfinavir (right). Red arrows indicate passages where virus was amplified, along with either sequencing of *gag* alone, *gag* and *protease*, or the full genome minus the LTRs (as indicated above the red arrows). (B) For each selection (PAV-206 versus nelfinavir), an HIV-1 genome map is shown, with positions of viral open reading frames (ORFs), 5′ LTR, and 3′ LTR, with a line below indicating their approximate nucleotide positions within the HIV-1 genome. In each case, ORFs in which dominant nonsynonymous mutations emerged during selection are color-coded as follows: *gag*, blue; *pro*, red; and *env*, brown. Beneath the HIV-1 genome maps, dominant nonsynonymous amino acid mutations (identified through sequencing of passages indicated by red arrows in panel A) are shown according to their position in the genome map at the top of panel B. The passage (P) in which these mutations were identified is indicated to the left of each genome map line. The amino acid mutations are color-coded according to the ORF in which they were found (colors described above). As indicated in the boxed legend, which applies to both panels, cyclophilin A binding loop mutations that have been previously identified as tissue culture adaptations are marked with a dagger symbol, and previously described nelfinavir-specific mutations are marked with an asterisk (with all references for these in the text and Tables 1 and 2).

The colocalization of PAV-818 with HIV-1 Gag suggested a straightforward model in which Gag is the target of this assembly inhibitor. If this is the case, then selection for resistance to the compound should result in rapid acquisition of compound-specific mutations in the *gag* gene, analogous to what has been observed for all antiretrovirals that target viral gene products (41). To determine whether development of resistance under selection could identify a viral target for this compound, we infected MT-2 T cells and passaged them under selection with PAV-206 for 37 weeks, increasing the drug concentration by 2-fold whenever evidence of resistance was observed and periodically sequencing virus to identify dominant mutations (Fig. 8A, sequencing events indicated by red arrows). We also selected with nelfinavir, a well-studied protease inhibitor, in parallel, as a positive control.

As would be expected, we observed that 37 passages in the presence of nelfinavir led to the emergence of three well known resistance mutations in HIV-1 protease: L10F, V77I, and L90M (indicated in red in Fig. 8B using HXB2 genome numbering; Table 1), with L90M being a primary resistance mutation and V77I and L10F being common secondary mutations that further boost nelfinavir resistance (42–46). L90M and V77I were first detected as dominant mutations at passage 29, just before the virus developed a high level of resistance, defined here as replication in a concentration of nelfinavir that is 16-fold higher than the EC_50_. L10F was detected later, becoming dominant at passage 37. In addition to these well described nonsynonymous protease-resistance mutations, two nonsynonymous substitutions in Gag emerged during nelfinavir selection (indicated in blue in Fig. 8B; Table 1). These substitutions, A224T and H219Q, are in the cyclophilin A binding loop of Gag and were detectable by passages 21 and 29, respectively. H219Q and other substitutions in the cyclophilin A binding loop (amino acids 217 to 225 in Gag) are polymorphisms in the Los Alamos Database (47, 48) that are known to modulate incorporation of cyclophilin A into virions when virus is passaged in cyclophilin-A-rich immortalized CD4^+^ T cells even in the absence of drugs, thereby resulting in virion cyclophilin A levels that are optimal for replication in these cell lines (49, 50). From these data, we infer that in the nelfinavir selection group, a tissue-culture-adaptive cyclophilin A binding loop polymorphism initially emerged in Gag and likely led to improved fitness and replication in a drug concentration 8-fold greater than the EC_50_; this was followed later by nelfinavir-specific resistance mutations, which likely led to high level nelfinavir resistance.

**TABLE 1:** Nonsynonymous mutations that arose upon selection in nelfinavir^*a*^

SC appearance^*b*^	Nelfinavir selection results				Notes
	Mutation no. relative to HXB2^*c*^	Mutation no. relative to NL4-3^*c*^	Frequency (%) of aa^*d*^	Mutation associated with:	
Dominant	**H219Q Gag** (H87Q p24 Gag)	H219Q Gag (H87Q p24 Gag)	H, 73.31 (6999); Q, 22.86 (2182); P, 1.73 (165)	Culture adaptation to intracellular CypA level (47, 49, 97); cyclosporine resistance (98)	In the cyclophilin A binding loop
≥50%	**A224T Gag** (A92T p24 Gag)	A224T Gag (A92T p24 Gag)	A, 67.49 (6443); P, 31.35 (2993)	Culture adaptation (99)	In the cyclophilin A binding loop
≥50%	**L10F protease** (L66F Pol)	L10F protease (L66F Pol)	L, 85.68 (4451); I, 7.10 (369); V, 5.47 (284)	Nelfinavir resistance (100); other PI resistance (45)	
Dominant	**V77I protease** (V133I Pol)	V77I protease (V133I Pol)	V, 83.10 (4322); I, 16.11 (838)	Nelfinavir resistance (42, 101)	
Dominant	**L90M protease** (L146M Pol)	L90M protease (L146M Pol)	L, 98.77 (5137); M, 1.15 (60)	Nelfinavir resistance (98); other PI resistance (45)	
≥50%	**S144R Env** (S114R gp120)	S144R Env (S114R gp120)	S, 24.14 (1248); N, 21.88 (1131); T, 16.12 (833)	Fusion inhibitor resistance (102)	In the Env V1 loop
Dominant	**A541V Env** (A30V gp41)	A539V Env (A30V gp41)	A, 94.84 (6728); V, 2.93 (208); T, 1.97 (140)	Culture adaptation (51, 54); Rev inhibitor resistance (103)	In the Rev response element
Dominant	**Q550H Env** (Q39H gp41)	Q548H Env (Q39H gp41)	Q, 99.17 (7035)	Culture adaptation (54); also arose in PAV-206 selection in present study	In the Rev response element; in the T-20 (Enfuvirtide) drug binding site

a Mutations are numbered relative to the HXB2 or NL4-3 genomes, with the frequency of the mutation, the phenotype associated with the mutation, and relevant references indicated.

b Gel sequencing chromatograms (SC) were visually inspected to identify a second chromatogram peak that is equal or larger in height than the peak corresponding to the reference sequence (defined here as ≥50%) or a single chromatogram peak that differs from the reference sequence (defined here as Dominant).

c Indicates viral protein to which numbering refers, either in the HXB2 or NL4-3 HIV strain. The mutation number relative to other relevant proteins or domains is indicated in parentheses. Mutations in boldface are shown in Fig. 8.

d Determined using AnalyzeAlign (available on Los Alamos HIV databases and compendiums site) using the premade Web Alignment; the top three that are >1% are listed. aa, amino acids. Entries are presented as follows: “amino acid, % frequency (number of isolates).”

Given the results with nelfinavir, we were surprised to find no dominant PAV-206-specific mutations in the *gag* or *pol* genes upon selection with PAV-206 in parallel. Notably, we did detect a dominant drug-independent, tissue-culture adaptive mutation in the cyclophilin A binding loop of Gag in the PAV-206 selection, as had been observed with nelfinavir. Specifically, at passage 19, we detected a V218M substitution in the cyclophilin A binding loop (Fig. 8; Table 2). Like the cyclophilin A binding loop substitutions observed with nelfinavir selection, V218M improves viral fitness in cyclophilin-A-rich CD4^+^ T cells through optimizing cyclophilin binding (47). Unlike with nelfinavir selection, no other dominant nonsynonymous mutations were observed in the *gag* or *pol* genes during 37 weeks of selection with PAV-206; moreover, high-level resistance was also not observed (Fig. 8), with an unsuccessful passage in a PAV-206 concentration that was 16-fold higher than the EC_50_ (J. Reed, unpublished observations).

**TABLE 2:** Mutations that arose upon selection in PAV-206^*a*^

SC appearance^*b*^	PAV-206 selection results				Notes
	Mutation no. relative to HXB2^*c*^	Mutation no. relative to NL4-3^*c*^	Frequency (%) of aa^*d*^	Mutation associated with:	
Nonsynonymous					
Dominant	**V218M Gag** (V86M p24 Gag)	V218M Gag (V86M p24 Gag)	V, 87.62 (8365); A, 4.85 (463); P, 3.40 (325)	Culture adaptation to intracellular CypA level (49, 50)	In the cyclophilin A binding loop
Dominant	**N302Y Env** (N272Y gp120)	N300Y Env (N270Y gp120)	N, 96._50_ (6844); K, 1.06 (75)	Culture adaptation – CCR5 (104–106); fusion inhibitor resistance (52)	In the Env V3 loop; involved in coreceptor binding
Dominant	**D547G Env** (D36G gp41)	D545G Env (D36G gp41)	G, 99.65 (7069)	Replication advantage (53, 104); fusion inhibitor resistance (107)	In the Rev response element; in the T-20 (Enfuvirtide) drug binding site; D36 in NL4-3 but G36 in most other strains of HIV-1 (19)
≥50%	**Q5_50_H Env** (Q39H gp41)	Q548H Env (Q39H gp41)	Q, 99.17 (7035)	Culture adaptation (54); also arose in nelfinavir selection in the present study	In the rev response element; in the T-20 (enfuvirtide) drug binding site
					
Synonymous					
≥50%	I_ATT_ 205 I_ATC_ Pol (I_ATT_ 50 I_ATC_ reverse trans.)	I_ATT_ 205 I_ATC_ Pol (I_ATT_ 50 I_ATC_ reverse trans.)		Unknown	
≥50%	Q_CAG_ 552 Q_CAA_ Env (Q_CAG_ 41 Q_CAA_ gp41)	Q_CAG_ 5_50_ Q_CAA_ Env (Q_CAG_ 41 Q_CAA_ gp41)		Replication advantage (108)	In the Rev response element; in the T-20 (Enfuvirtide) drug binding site
Dominant	V_GTG_ 583 V_GTA_ Env (V_GTG_ 72 V_GTA_ gp41)	V_GTG_ 581 V_GTA_ Env (V_GTG_ 72 V_GTA_ gp41)		Fusion inhibitor resistance (109)	In the Rev response element

a Mutations are numbered relative to the HXB2 or NL4-3 genomes, with the frequency of the mutation, the phenotype associated with the mutation, and relevant references indicated.

b Gel sequencing chromatograms (SC) were visually inspected to identify a second chromatogram peak that is equal or larger in height than the peak corresponding to the reference sequence (defined here as ≥50%) or a single chromatogram peak that differs from the reference sequence (defined here as Dominant).

c Indicates viral protein to which numbering refers, either in the HXB2 or NL4-3 HIV strain. Numbering of mutation relative to other relevant proteins or domains is indicated in parentheses. Mutations in boldface are shown in Fig. 8.

d Determined using AnalyzeAlign (available on Los Alamos HIV databases and compendiums site) using the premade Web Alignment; the top three that are >1% are listed. aa, amino acids. Entries are presented as follows: “amino acid, % frequency (number of isolates).”

While our primary interest was in dominant nonsynonymous mutations in *gag* or *pol*, which include the most plausible targets of PAV-206, we also looked for mutations elsewhere in the HIV-1 genome. Interestingly, nonsynonymous mutations in *env* were observed in both selection groups (A541V, Q550H, and S144R in the nelfinavir selection group and N302Y, D547G, and Q550H in the PAV-206 selection group, indicated in brown in Fig. 8; Tables 1 and 2). These mutations are known to confer global replication advantage or serve as tissue culture adaptations. Specifically, A541V (observed at nelfinavir passage 29) was recently shown to confer broad escape from defects in virus replication caused by either virus mutations or antiretroviral drugs, most likely by increasing cell-to-cell transmission in T cell lines (51). Similarly, N302Y and D547G (observed at PAV-206 passage 27) are thought to increase fusion kinetics (52) and enhance fusogenicity (53), respectively, and likely confer global replication advantages. Q550H, which arose during both PAV-206 and nelfinavir selection, has been observed in the absence of drug treatment (54) and is therefore likely to be a tissue culture adaptation. Thus, these *env* mutations are not specific to PAV-206 selection. Finally, we also observed three synonymous mutations arising in *env* upon PAV-206 selection (Table 2). Overall, we concluded from these resistance studies that replication in PAV-206 for 37 weeks failed to select for any PAV-206-specific resistance mutations in *gag* or *pol* and therefore differed markedly from the nelfinavir selection control, which demonstrated the classic pattern of multiple resistance mutations emerging in the viral target of the drug. Consistent with this conclusion, we observed a high level of resistance in the nelfinavir selection group at passage 33 that correlated with emergence of nelfinavir-specific mutations in *pro*, while resistance to PAV-206 failed to rise above the moderate level that is likely due to tissue culture adaptation and mutations that confer global replication advantage in T cells (Fig. 8A).

### The biotinylated antiretroviral PAV-206 analog colocalizes with two host components of assembly intermediates, suggesting a host-targeting mechanism.

**FIG 9:**
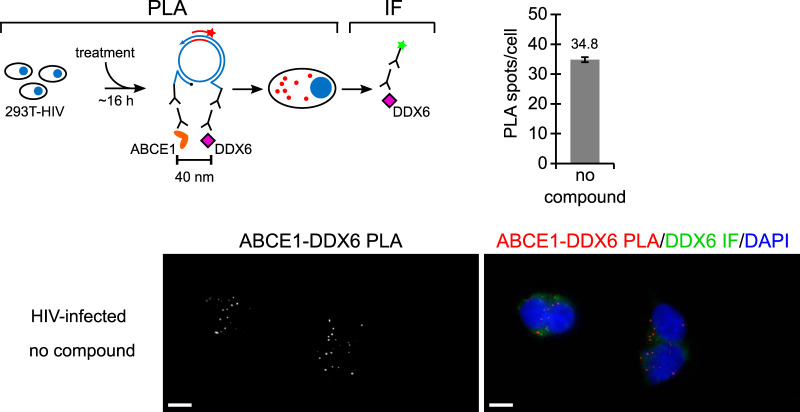
Two markers of HIV-1 assembly intermediates, ABCE1 and DDX6, are colocalized in untreated HIV-1-expressing cells. The schematic shows the PLA approach for detecting colocalization of ABCE1 with DDX6. 293T cells chronically infected with HIV-1 (293T-HIV) but not treated with any compounds were analyzed by PLA, as described in the Fig. 6 legend, except that the primary antibodies used were rabbit anti-ABCE1 and mouse anti-DDX6, with red spots representing sites where ABCE1 and DDX6 are colocalized *in situ*. The graph shows the average number of ABCE1-DDX6 spots per cell. Ten fields were analyzed (containing a total of 121 cells), with error bars showing the SEM. A representative field is shown, with the image on the left displaying ABCE1-DDX6 PLA spots alone in grayscale, and the image on the right displaying a merge of three color channels: ABCE1-DDX6 PLA (red), DDX6 IF (green), and DAPI-stained nuclei (blue).

Our studies to this point had shown that the PAV-206 chemotype colocalizes with Gag *in situ* (Fig. 6) and appears to inhibit Gag assembly (Fig. 4) but, unexpectedly, does not appear to target Gag or GagPol based on resistance studies (Fig. 8). This led us to hypothesize that PAV-206 and its analogs colocalize with host proteins, as well as Gag, which would raise the possibility that PAV-206 targets a host protein or virus-host interface that is critical for assembly. This possibility is made more plausible by the fact that these compounds were identified through assembly screens that recapitulate host-catalyzed capsid assembly pathways. In the case of HIV-1 immature capsid assembly, two host proteins, ABCE1 and DDX6, are known to promote assembly and are associated with Gag by coimmunoprecipitation (co-IP), serving as markers of Gag-containing assembly intermediates (26, 30–34). Previously, we have shown that ABCE1 colocalizes with Gag, as does DDX6 (24); here, we also used PLA to show that ABCE1 and DDX6 are colocalized in HIV-1-infected cells (Fig. 9), as would be expected for markers of assembly intermediates. Thus, we next used PLA to ask whether PAV-206 colocalizes with these Gag-associated host proteins.

**FIG 10:**
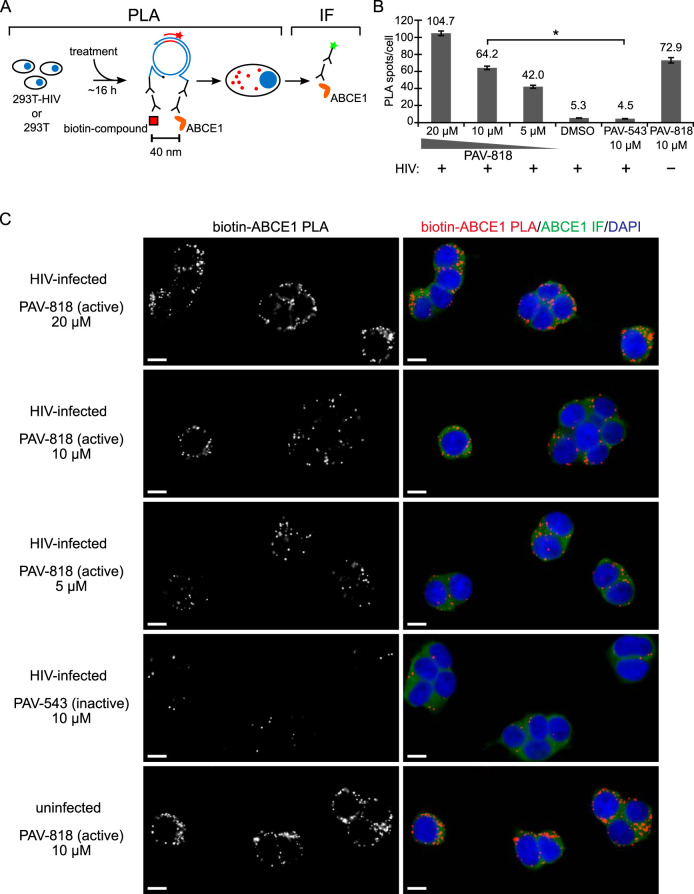
The biotinylated antiretroviral PAV-206 analog colocalizes *in situ* with ABCE1, a host component of assembly intermediates. (A) Schematic of the PLA approach for detecting colocalization of compound with ABCE1. 293T cells chronically infected with HIV-1 (293T-HIV) or uninfected 293T cells were treated with indicated amounts of PAV-818 (the biotinylated active compound), PAV-543 (the biotinylated inactive compound), or DMSO for 16 h. PLA was performed by incubation with primary antibodies (mouse anti-biotin and rabbit anti-ABCE1), followed by PLA secondary antibodies and other reagents as described in Fig. 6. Red spots indicate sites where biotinylated compound and ABCE1 are colocalized *in situ*. After PLA, IF was performed (green star) to mark intracellular ABCE1 with low-level green fluorescence. (B) The graph shows the average number of biotin-ABCE1 PLA spots per cell for each condition, with “+” indicating HIV-1-infected cells and “–” indicating uninfected cells. Ten fields were analyzed for each group (containing a total of 104 to 155 cells per group), with error bars showing the SEM. *, Significant difference in the number of biotin-ABCE1 PLA spots per cell when comparing treatment with PAV-818 versus PAV-543, both at 10 μM (*P* < 0.001). (C) A representative field for each group quantified in panel B is shown, except for DMSO treatment. Fields on the left show biotin-ABCE1 PLA spots alone in grayscale. To the right are the same fields shown as a merge of three color channels: biotin-ABCE1 PLA (red), ABCE1 IF (green), and DAPI-stained nuclei (blue). Scale bars, 10 μm.

293T cells chronically infected with HIV-1 were treated with either the biotinylated antiretroviral compound PAV-818, the biotinylated but inactive compound PAV-543, or DMSO and then subjected to biotin-ABCE1 PLA (Fig. 10A). Quantification of red fluorescent spots representing sites where biotinylated compound and ABCE1 are colocalized *in situ* revealed 14.3-fold more spots in cells treated with the antiviral PAV-818 (10 μM) than in cells treated with an equivalent concentration of PAV-543 and a similar difference relative to DMSO treatment (Fig. 10B and C). In addition, the number of biotin-ABCE1 spots observed with PAV-818 treatment displayed a dose-response relationship (Fig. 10B and C). Interestingly, PAV-818 colocalized with ABCE1 as strongly in uninfected 293T cells as in HIV-1-infected human cells (Fig. 10B and C). As expected, controls in which antibodies to biotin or to ABCE1 were individually replaced with an NI control antibody generated very few PLA spots (Fig. 7B).

**FIG 11:**
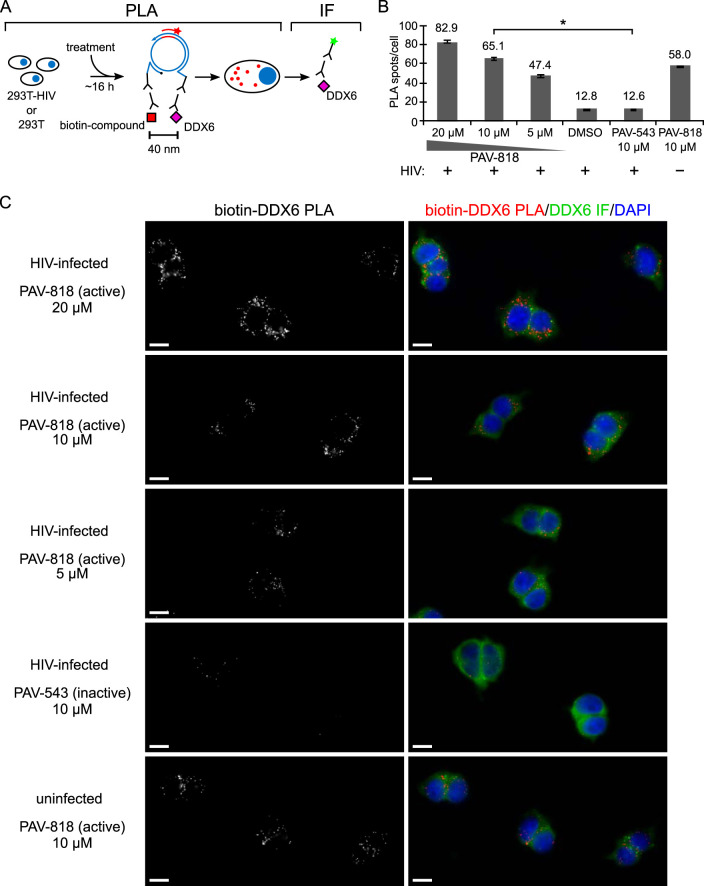
The biotinylated antiretroviral PAV-206 analog colocalizes *in situ* with DDX6, an RNA granule protein in assembly intermediates that colocalizes with ABCE1. (A) Schematic of the PLA approach for detecting colocalization of compound with DDX6. 293T cells chronically infected with HIV-1 (293T-HIV) or uninfected 293T cells were treated with indicated amounts of PAV-818 (the biotinylated active compound), PAV-543 (the biotinylated inactive compound), or DMSO for 16 h. PLA was performed by incubation with primary antibodies (mouse anti-biotin and rabbit anti-DDX6), followed by PLA secondary antibodies and other reagents as described in Fig. 6. Red spots indicate sites where biotinylated compound and DDX6 are colocalized *in situ*. After PLA, IF was performed (green star) to mark intracellular DDX6 with low-level green fluorescence. (B) The graph shows the average number of biotin-DDX6 PLA spots per cell for each condition, with “+” indicating HIV-1-infected cells and “–” indicating uninfected cells. Five fields were analyzed for each group (containing a total of 39 to 73 cells per group), with error bars showing the SEM. *, Significant difference in the number of biotin-DDX6 PLA spots per cell when comparing treatment with PAV-818 versus PAV-543, both at 10 μM (*P* < 0.001). (C) A representative field for each group quantified in panel B is shown, except for DMSO treatment. Fields on the left show biotin-DDX6 PLA spots alone in grayscale. To the right are the same fields shown as a merge of three color channels: biotin-DDX6 PLA (red), DDX6 IF (green), and DAPI-stained nuclei (blue). Scale bars, 10 μm.

PAV-818 was also found to colocalize with DDX6 in chronically HIV-1-infected 293T cells by PLA in a dose-dependent manner, with ∼5.1-fold more spots observed in PAV-818-treated cells than in PAV-543-treated or DMSO-treated cells (Fig. 11; NI controls in Fig. 7B). Notably, PAV-818 colocalized with DDX6 as strongly in uninfected 293T cells as in HIV-1-infected human cells (Fig. 11B and C), as was observed for PAV-818-ABCE1 colocalization (Fig. 10B and C). Thus, this novel antiretroviral compound colocalizes with HIV-1 Gag and with the host proteins ABCE1 and DDX6, all of which are components of HIV-1 assembly intermediates, and colocalizes with ABCE1 and DDX6 even in the absence of HIV-1 infection.

### The biotinylated antiretroviral PAV-206 analog does not colocalize with two other host RNA granule proteins, suggesting selectivity.

The colocalization of the biotinylated antiretroviral PAV-206 analog with two host proteins present in assembly intermediates raised the possibility that this compound colocalizes nonspecifically with host proteins. To address this concern, we sought to determine whether this biotinylated analog colocalizes with host RNA granule proteins that have not been found associated with HIV-1 Gag by co-IP to date. For this purpose, we chose G3BP1 and XRN1, which are largely found in stress granules and P-bodies, respectively (reviewed in reference 55).

**FIG 12:**
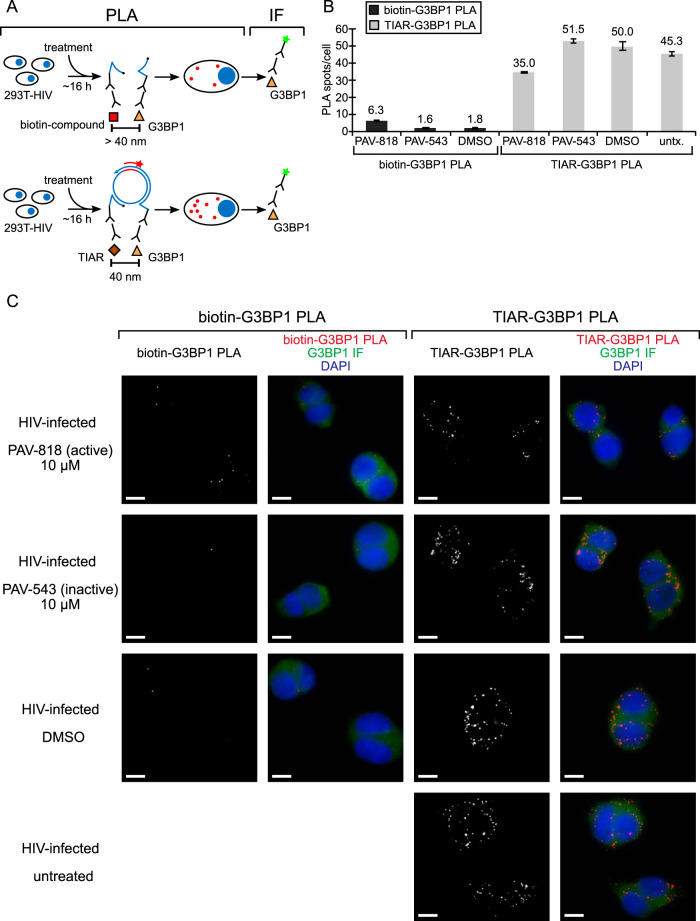
The biotinylated antiretroviral PAV-206 analog does not colocalize *in situ* with G3BP1, an RNA granule protein in stress granules. (A) Schematic of the PLA approaches for detecting colocalization of compound with G3BP1 and colocalization of G3BP1 with TIAR, two known stress granule proteins, are shown. 293T cells chronically infected with HIV-1 (293T-HIV) were treated with 10 μM PAV-818 (the biotinylated active compound), 10 μM PAV-543 (the biotinylated inactive compound), or DMSO for 16 h. The upper diagram depicts biotin-G3BP1 PLA performed by incubation with primary antibodies (mouse anti-biotin and rabbit anti-G3BP1), followed by PLA secondary antibodies and other reagents as described in Fig. 6. Red spots indicate sites where biotinylated compound and G3BP1 are colocalized *in situ*. The lower diagram depicts TIAR-G3BP1 PLA performed by incubation with primary antibodies (mouse anti-TIAR and rabbit anti-G3BP1), followed by PLA secondary antibodies and reagents as described in Fig. 6. Red spots indicate sites where the two stress granule proteins TIAR and G3BP1 are colocalized *in situ*. For upper and lower panels, following PLA, IF was performed (green star) to mark intracellular G3BP1 with low-level green fluorescence. (B) Left side of graph (dark gray bars) shows the average number of biotin-G3BP1 PLA spots per cell for each condition with representative images shown in panel C at the left. Five fields were analyzed for each group (containing a total of 63 to 83 cells per group), with error bars showing the SEM. The right side of the graph (light gray bars) shows the average number of TIAR-G3BP1 PLA spots per cell for each condition, with representative images shown in panel C at the right. Five fields were analyzed for each group (containing a total of 63 to 75 cells per group), with error bars showing the SEM. (C) A representative field for each group quantified in the graph is shown. The two leftmost columns of images display biotin-G3BP1 PLA spots alone in grayscale (first column) and a merge of three color channels (second column): biotin-G3BP1 PLA (red), G3BP1 IF (green), and DAPI-stained nuclei (blue). The two rightmost columns of images display TIAR-G3BP1 PLA spots alone in grayscale (third column) and a merge of three color channels (fourth column): TIAR-G3BP1 PLA (red), G3BP1 IF (green), and DAPI-stained nuclei (blue). Scale bars, 10 μm.

**FIG 13:**
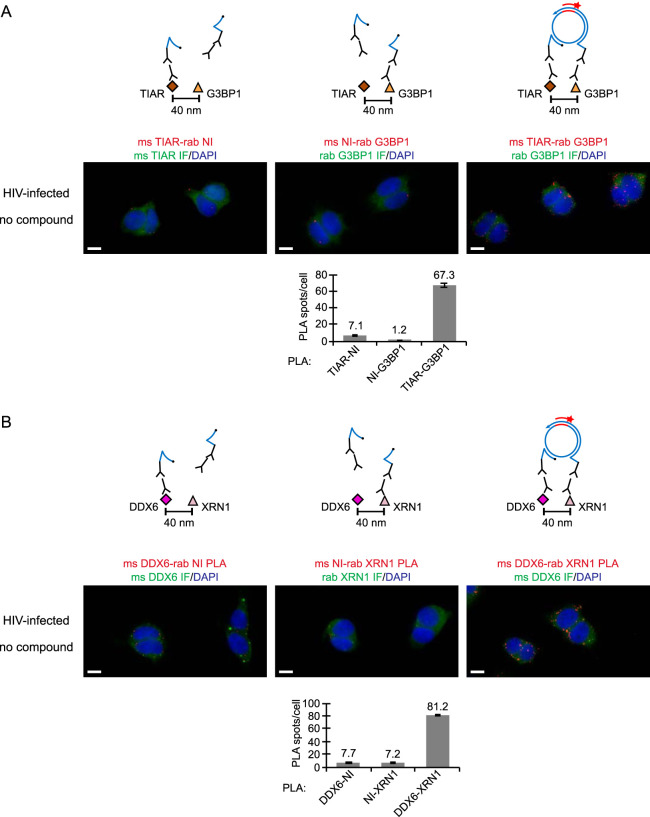
Nonimmune controls for the TIAR-G3BP1 and DDX6-XRN1 proximity ligation assays. (A) NI controls for the TIAR-G3BP1 PLA. Above each PLA image is the schematic corresponding to the PLA approach in that panel. Untreated 293T cells chronically infected with HIV-1 (293T-HIV) were analyzed by PLA. For the positive control (image and schematic on the right), PLA was performed by incubating with primary antibodies, mouse anti-TIAR and rabbit anti-G3BP1, followed by PLA secondary antibodies and other reagents as described in Fig. 6. In the two negative controls (images and schematics at left and center), one primary antibody was replaced with an NI control antibody from the same species, as indicated. Red spots indicating colocalization of the TIAR with G3BP should be absent when either primary antibody is replaced by an NI antibody. After PLA, TIAR or G3BP IF was performed to mark host protein-expressing cells with low-level green fluorescence. Images show a representative field for each of the three antibody pairs. Fields are shown as a merge of three color channels: the red channel shows TIAR-G3BP1 PLA, TIAR-NI PLA, or NI-G3BP1 PLA, as indicated by red labeling above images; the green channel shows TIAR IF or G3BP1 IF as indicated by green labeling above images; and the blue channel shows DAPI-stained nuclei. Scale bars, 10 μm. Graph below shows the average number of PLA spots per cell for each antibody pair. Five fields were analyzed for each group (containing a total of 45 to 62 cells per group), with error bars showing the SEM. (B) NI controls for the DDX6-XRN1 PLA. Above each PLA image is the schematic corresponding to the to the PLA approach in that panel. Untreated 293T cells chronically infected with HIV-1 (293T-HIV) were analyzed by PLA. For the positive control (image and schematic on the right), PLA was performed by incubating with primary antibodies, mouse anti-DDX6 and rabbit anti-XRN1, followed by PLA secondary antibodies and other reagents as described in Fig. 6. In the two negative controls (images and schematics at left and center), one primary antibody was replaced with an NI control antibody from the same species, as indicated. Red spots indicating colocalization of the DDX6 with XRN1 should be absent when either primary antibody is replaced by a NI antibody. Following PLA, DDX6 or XRN1 IF was performed to mark host protein-expressing cells with low-level green fluorescence. Images show a representative field for each of the three antibody pairs. Fields are shown as a merge of three color channels: the red channel shows DDX6-XRN1 PLA, DDX6-NI PLA, or NI-XRN1 PLA, as indicated by red labeling above images; the green channel shows DDX6 IF or XRN1 IF, as indicated by green labeling above images; and the blue channel shows DAPI-stained nuclei. Scale bars, 10 μm. Graph below shows the average number of PLA spots per cell for each antibody pair. Five fields were analyzed for each group (containing a total of 46 to 73 cells per group), with error bars showing the SEM.

PLA analysis revealed that the biotinylated antiretroviral compound (PAV-818) does not colocalize with the stress granule protein G3BP1 (Fig. 12). To confirm that the G3BP1 antibody did indeed detect G3BP1 in our PLA experiment, we included a positive control in which we demonstrated that G3BP1 colocalizes with the stress granule protein TIAR by PLA regardless of compound treatment (Fig. 12), as would be expected since both proteins are found in stress granules (56). NI controls confirmed that both antibodies are required for PLA signal (Fig. 13A).

**FIG 14:**
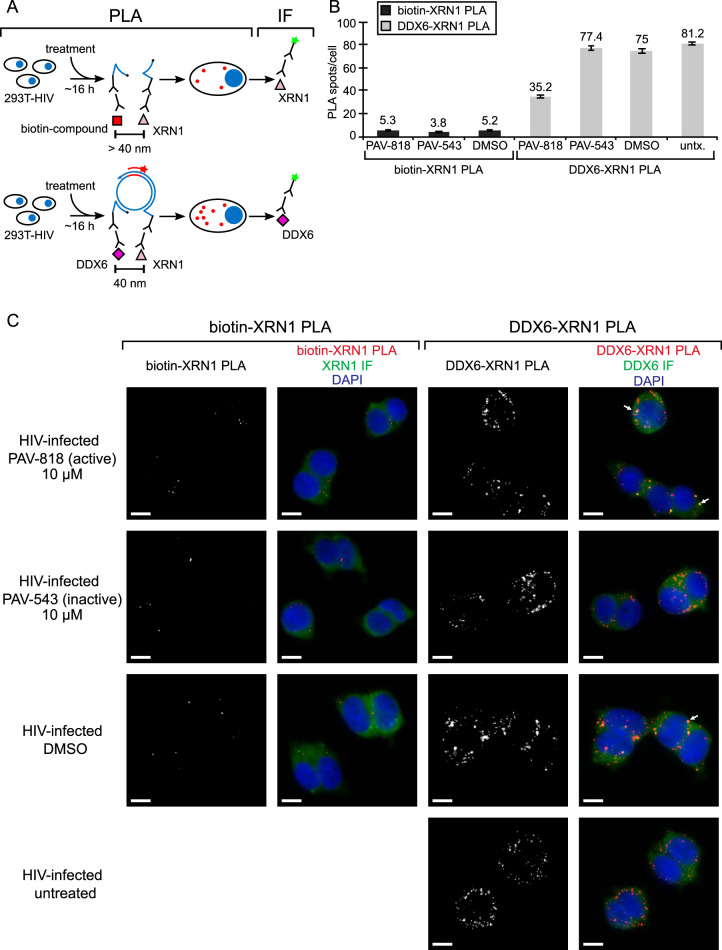
The biotinylated antiretroviral PAV-206 analog does not colocalize *in situ* with XRN1, a host enzyme found in P-bodies. (A) Schematic of the PLA approaches for detecting colocalization of compound with XRN1 and colocalization of XRN1 with DDX6, both known to be present in P-bodies, are shown. 293T cells chronically infected with HIV-1 (293T-HIV) were treated with 10 μM PAV-818 (the biotinylated active compound), 10 μM PAV-543 (the biotinylated inactive compound), or DMSO for 16 h. The upper diagram depicts biotin-XRN1 PLA performed by incubation with primary antibodies (mouse anti-biotin and rabbit anti-XRN1), followed by PLA secondary antibodies and reagents as described in Fig. 6. Red spots indicate sites where biotinylated compound and XRN1 are colocalized *in situ*. After PLA, IF was performed (green star) to mark intracellular XRN1 with low-level green fluorescence. The lower diagram depicts DDX6-XRN1 PLA performed by incubation with primary antibodies (mouse anti-DDX6 and rabbit anti-XRN1), followed by PLA secondary antibodies as described as described in Fig. 6. Red spots indicate sites where the two P-body proteins DDX6 and XRN1 are colocalized *in situ*. After PLA, IF was performed (green star) to mark intracellular DDX6 with low-level green fluorescence. (B) The left side of the graph (dark gray bars) shows the average number of biotin-XRN1 PLA spots per cell for each condition, with representative images shown in panel C at the left. Five fields were analyzed for each group (containing a total of 44 to 54 cells per group), with error bars showing the SEM. The right side of the graph (light gray bars) shows the average number of DDX6-XRN1 PLA spots per cell for each condition, with representative images shown in panel C at the right. Five fields were analyzed for each group (containing a total of 57–73 cells per group), with error bars showing the SEM. (C) A representative field for each group quantified in the graph is shown. The two leftmost columns of images display biotin-XRN1 PLA spots alone in grayscale (first column) and a merge of three color channels (second column): biotin-XRN1 PLA (red), XRN1 IF (green), and DAPI-stained nuclei (blue). The two rightmost columns of images display DDX6-XRN1 PLA spots alone in grayscale (third column) and a merge of three color channels (fourth column): DDX6-XRN1 PLA (red), DDX6 IF (green), and DAPI-stained nuclei (blue). White arrows show sites where DDX6-XRN1 PLA signal colocalizes with large DDX6-IF-positive structures that are likely to be P-bodies. Scale bars, 10 μm.

Finally, we also showed that the biotinylated antiretroviral compound (PAV-818) does not colocalize with the P-body marker XRN1 (Fig. 14). To confirm that the XRN1 antibody did indeed detect XRN1 in our PLA experiment, we included a positive control in which we demonstrated that XRN1 colocalizes with DDX6 by PLA regardless of compound treatment (Fig. 14; with NI controls in Fig. 13B), as would be expected since both proteins are found in P-bodies (reviewed in reference 57). In this PLA experiment, we also performed indirect immunofluorescence (IF) analysis with antibody to DDX6 (following PLA) to determine whether XRN1-DDX6 PLA signal is detected in P-bodies (Fig. 14C). Indeed, we observed that, in treated and untreated cells, some of the DDX6-XRN1 PLA signal colocalized with large DDX6-IF-positive spots that likely correspond to P-bodies (Fig. 14C, white arrows pointing to large yellow spots in merged panels), suggesting colocalization of these two proteins in P-bodies, as would be expected. Notably, we demonstrated previously that assembly intermediates, which contain DDX6, are distinct from and much smaller than P-bodies, which also contain DDX6 (24). Thus, together the PLA data suggest that the biotinylated antiretroviral compound associates selectively with an ABCE1- and DDX6-containing RNP complex that Gag coopts to form HIV-1 assembly intermediates and does not associate with P-bodies, which likely contain a separate pool of DDX6.

## DISCUSSION

**FIG 15:**
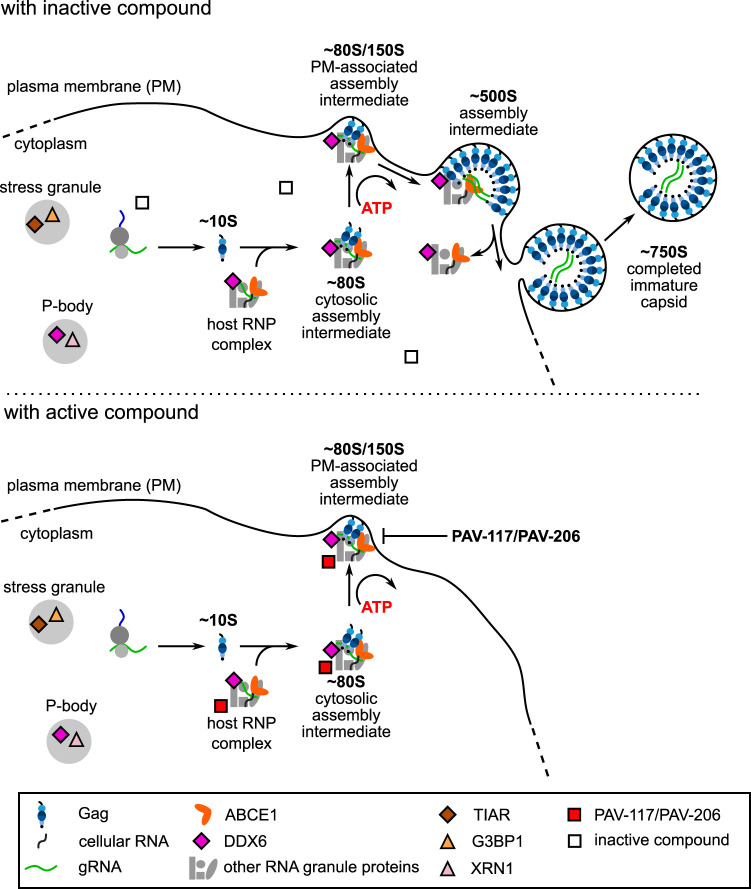
Model for the action of PAV-206, a possible first-in-class selective inhibitor of HIV-1 assembly. Upper diagram shows the intracellular pathway for HIV-1 immature capsid assembly, in the presence of an inactive compound, starting with synthesis of HIV-1 Gag and formation of the early ∼10S assembly intermediate. The ∼80S assembly intermediate is formed when HIV-1 Gag coopts a host RNP complex related to an RNA granule to form the ∼80S cytosolic intermediate. This and subsequent intermediates contain Gag associated with ABCE1, DDX6, other host factors, and HIV-1 gRNA. After targeting of the cytosolic ∼80S assembly intermediate to the host plasma membrane, Gag multimerization continues leading to the formation of the ∼500S assembly intermediate. The host proteins are released upon formation of the ∼750S completed immature capsid, and budding results in release of the completed immature capsid. The lower diagram shows one model for PAV-117/PAV-206 activity based on the findings in this study. Our colocalization studies suggest that this chemotype is associated with both the HIV-1 assembly intermediates and the host RNA-granule-related complex from which they are derived, since the compound colocalizes with two host components of this complex, ABCE1 and DDX6, in the presence and absence of Gag. The compound does not colocalize with G3BP1 or XRN1, proteins found in stress granules and P-bodies, indicating that this chemotype acts with some selectivity for the RNP complex that is coopted to form assembly intermediates. In addition, we observed a reduction of virus production and the amount of ∼500S assembly intermediate in PAV-117-treated infected cells. Thus, we hypothesize that PAV-117/PAV-206 inhibits virus production by acting during the HIV-1 immature capsid assembly pathway to inhibit progression of assembly past the ∼80S/150S assembly intermediate.

Here, we report our discovery of a family of small molecules, PAV-206 and its analogs, that potently inhibit HIV-1 replication by acting on intracellular viral late events, a stage of the viral life cycle that is not specifically targeted by potent inhibitors currently in use or reported to be in development. While the exact target and mechanism of action of this compound remain to be determined, data from virologic, resistance, and imaging studies presented here suggest that this chemotype does not target a viral protein but instead may target a component of a host multiprotein complex that plays an important role in assembly of the immature HIV-1 capsid (Fig. 15). While further studies will be needed to test this hypothesis, PAV-206 and its analogs should be of great interest even at this early stage given that all antiretroviral drugs in use except one (CCR5 antagonists) target viral proteins and that host-targeting antivirals offer a high genetic barrier to resistance that will be key as resistance to existing drugs becomes more prevalent (58, 59).

Most exciting in this regard was our observation that after selection with increasing concentrations of PAV-206 for 37 weeks in MT-2 T cells, sequencing of replicating virus revealed no compound-specific mutations in *gag* or *pol* (Fig. 8), suggesting that PAV-206 acts by a mechanism that is independent of direct targeting of Gag. Consistent with this conclusion, high-level resistance to PAV-206 was not observed during this time (Fig. 8). Such behavior is distinctly unusual, since all existing classes of direct-acting antiretroviral drugs result in rapid development of high-level resistance due to mutations in their viral targets upon single drug treatment (41), with the exception of integrase strand transfer inhibitors, which are less prone to the development of high-level resistance (60). The unusual nature of this finding was emphasized by our contrasting finding that 37-week selection with the protease inhibitor nelfinavir in parallel did result in three known resistance mutations in the HIV-1 protease, as well as high-level resistance (allowing growth in nelfinavir concentrations 16× higher than the EC_50_), as would be expected.

We did observe two other categories of mutations during both PAV-206 and nelfinavir selection. The first category of these compound-independent mutations comprises known tissue-culture adaptations (Fig. 8; Tables 1 and 2). Most of these were in the cyclophilin A loop of Gag and are known to reduce the amount of cyclophilin A incorporated into virions during passage in cyclophilin A-rich immortalized CD4^+^ T cells, in the presence or absence of antiretroviral drugs (49, 50). Another known tissue culture adaptation (54) was observed in *env* (Q550H) and also arose in both selection groups. A second category of compound-independent mutations likely confers a more global replication advantage, as best demonstrated for A541V (51). The modest resistance observed relatively early in the PAV-206 selection experiment (allowing virus replication in a PAV-206 concentration 8× higher than the EC_50_) might be explained by the early, subdominant emergence of the cyclophilin A binding loop mutation V218M, a tissue culture adaptation. In support of this possibility, the appearance of modest resistance (to 8× EC_50_) in the nelfinavir selection control by passage 20 also correlated with emergence of a cyclophilin A binding loop polymorphism, A224T, and preceded the time when the drug-specific protease mutations became dominant. Further studies will be needed to test the hypothesis that these polymorphisms allow low level replication in the presence of compound.

While additional studies will be required to identify the target of PAV-206, the surprising lack of compound-specific viral resistance mutations in *gag* or *pol* raises the possibility that this compound acts on a host protein. Two other observations are consistent with that hypothesis. First, this compound was identified using novel screens that recapitulate host-catalyzed pathways in which assembling capsid proteins form a series of host-protein containing assembly intermediates, culminating in formation of the completed immature capsid. Such an assembly pathway screen was previously shown to identify a host-targeting small molecule that potently inhibits RABV in cell culture (35). Second, our imaging studies show that PAV-206 colocalizes with Gag, as well as ABCE1 and DDX6 (Fig. 6, 9, and 11), two host enzymes known to be associated with Gag in assembly intermediates, as shown by PLA and co-IP (24–27, 30, 32–34); here, we additionally showed that ABCE1 colocalizes with DDX6 by PLA (Fig. 9), consistent with previous co-IP results (30). Interestingly, our studies found that PAV-206 colocalizes with both of these host enzymes, ABCE1 and DDX6, in the absence of HIV-1 infection (Fig. 10 and 11), suggesting that the compound acts on the host RNA-granule-related RNP complex that is the precursor of assembly intermediates, even in the absence of Gag. Additional studies will be required to identify the direct target of this chemotype through cross-linking approaches that utilize the diazirine group in PAV-818, but based on data presented here we hypothesize that the target may be a component of the host-RNP-complex-derived assembly intermediates.

Our studies also demonstrate that the PAV-206 analogs associate with specific RNP complexes. We found that the PAV-206 analog does not associate with the host RNP G3BP1, a marker for stress granules, or XRN1, a marker for P-bodies (Fig. 12 and 14), neither of which are found in HIV-1 assembly intermediates. As positive controls, we showed that in the same experiments G3BP1 colocalizes with another stress granule protein (TIAR) and XRN1 colocalizes with another P-body protein (DDX6). While these positive-control colocalizations were robust in the absence and presence of compounds, the amount of colocalization (number of PLA spots) was lower upon treatment with the PAV-206 analog in both cases (Fig. 12B and 14B), suggesting that the antiretroviral compound affects host RNP dynamics, albeit without significant cellular toxicity. Notably, our data also suggest that the PAV-206 analog selectively recognizes a subset of intracellular complexes containing the RNP DDX6 (Fig. 14). Previously, we showed that the pool of DDX6 in P-bodies is distinct from the pool of DDX6 in much smaller RNPs that likely correspond to the precursors of assembly intermediates (24). Together, these data suggest that the PAV-206 analogs do not associate with P-bodies, which contain DDX6 and XRN1, but do associate with a host RNP that contains DDX6 but not XRN1. Thus, given our findings that PAV-206 selectively colocalizes with three proteins found in assembly intermediates (Gag, ABCE1, and DDX6) and that the PAV-206 analog reduces levels of the ∼500S assembly intermediate, we favor a model in which PAV-206 analogs selectively associate with a specific host RNP that is coopted to form assembly intermediates.

PAV-206 and its analogs are also of great interest because, to our knowledge, they are the first potent small molecules shown to selectively inhibit late events in the HIV-1 life cycle. Unlike previously reported compounds that target CA with high potency (i.e., EC_50_ in the nM range or below, e.g., GS-CA1 [12, 17, 61–63]), the chemotype reported here has no effects on viral early events and acts entirely on late events (Fig. 3). Moreover, PAV-117 reduces virus production (Fig. 3 and 4), suggesting that it acts on intracellular late events. PAV-117 treatment of infected cells did not reduce steady-state levels of two cellular proteins, suggesting that PAV-117 does not globally inhibit protein synthesis, as would be expected based on the screening strategy. PAV-117 treatment did lead to a reduction in steady-state intracellular Gag levels, which in turn is likely to reduce capsid assembly since assembly is dependent on intracellular Gag concentration (64). It is unlikely that PAV-117 reduces steady-state intracellular Gag levels by specifically inhibiting Gag synthesis, given its lack of effect on steady-state levels of other proteins. Instead, our data suggest an alternate hypothesis for how PAV-117 reduces steady-state Gag levels—that association of PAV-117 with assembly intermediates results in the degradation of these intermediates, leading to a reduction in steady-state Gag levels and immature capsid assembly. In support of our hypothesis that PAV-117 reduces intracellular Gag levels by acting posttranslationally on Gag-containing assembly intermediates, we observed a significant reduction in the relative level of the final (∼500S) assembly intermediate upon treatment with PAV-117 (Fig. 4C, 0.75 μM). Nevertheless, while the data suggest that the compound acts on a specific assembly intermediate (Fig. 15), additional studies are needed to determine the exact mechanism of action of PAV-206 and its analogs.

Finally, it should be noted that discovery of these compounds constitutes a compelling validation of the importance of HIV-1 RNA-granule-related assembly intermediates in virus production. Numerous groups in addition to ours have implicated RNA granule proteins as being important for assembly of diverse retroviruses and retroelements. For example, knockout studies demonstrated that RNA granule proteins are required for Ty3 retrotransposition in yeast and likely act during assembly (65–67). In addition studies showed that the RNA granule protein DDX6 is required for foamy virus packaging (68), and the RNA granule protein Stau1 plays an important role in HIV-1 Gag assembly and packaging (69–72). Finally, the RNA granule protein MOV10 has been shown to influence immature HIV-1 assembly (73, 74). Data presented here shows that the study of host-RNA-granule-derived HIV-1 assembly intermediates has led to discovery of the first compounds that specifically inhibit intracellular HIV-1 late events. Thus, these data add strong support to the view that such assembly intermediates not only exist but are highly relevant to our understanding of HIV-1 assembly, as the well as development of antiretroviral compounds for treatment of multidrug-resistant HIV-1 in the future.

## MATERIALS AND METHODS

### Cell-free protein synthesis and assembly reactions.

Cell-free capsid assembly reactions for HIV-1, VEEV, FLUV, HCV, DENV, RVFV, HNTV, and RPXV used wheat germ extract and were programmed with capsid protein-encoding mRNA transcript using methods analogous to those previously described for HIV-1 Gag (28) and HCV Core protein (75, 76) but without radiolabeling (all amino acids were unlabeled). Reaction mixtures were incubated at 26°C for 20 to 90 min and, in some cases, with a subsequent incubation at 34°C, which was optimal for the assembly of some viral capsids. For the HIV-1, VEEV, FLUV, HCV, DENV, RVFV, and HNTV reactions, the capsid protein mRNA transcripts that were used encoded HIV-1 Gag, VEEV core protein, FLUV nucleoprotein, HCV core protein, DENV capsid protein, RVFV nucleoprotein, and HNTV nucleoprotein, respectively.

### Description of the capsid assembly plate assay.

Cell-free capsid protein assembly reactions are programmed with transcript encoding the relevant capsid protein in 384-well plates in the presence of small molecules from a compound library. Upon completion of assembly, reactions are transferred to separate plates coated with anti-capsid protein antibody (capture antibody), which immobilizes the cell-free capsid assembly product. Captured capsid protein is detected with a biotinylated detection antibody, which in turn is recognized by avidin-horseradish peroxidase (HRP). Addition of a fluorogenic HRP substrate results in generation of a fluorescent signal whose intensity is expected to be proportional to the number of capsid epitopes displayed by the particles immobilized by capture antibody. Because of its multimeric nature, properly formed capsid-like particles are expected to display many capsid protein epitopes at each capture site and should therefore generate a large signal; a smaller signal would be expected from reactions performed in the presence of drugs that either inhibit capsid protein multimerization or alter capsid protein epitopes so that they are no longer effectively recognized by the capture or detection antibody. Thus, the plate assay fluorescent readout is expected to quantitatively indicate the amount of assembled capsid-like particles that are present in each well’s cell-free protein synthesis and assembly reaction. In addition, to exclude drugs that simply inhibit protein synthesis, GFP mRNA is included in parallel with capsid protein mRNA. The resulting GFP is inert with regard to capsid assembly but serves as an indicator of nonspecific inhibition of protein synthesis and can be quantified prior to transfer of cell-free translation products to the capture plate. Only compounds that inhibited the capsid-specific antibody signal without similar effects on GFP fluorescence were pursued further.

### Generation of antibodies for viral capsid assembly plates.

Peptides were chosen from the major capsid protein that were likely to represent immunogenic epitopes displayed on the surface of the viral capsid, for each viral family of interest, with three exceptions, FLUV, DENV, and RVFV, where recombinant protein to the entire major capsid protein/nucleoprotein was used as the immunogen. For peptide antisera, peptides were synthesized with a cysteine added at either the N or C terminus for coupling and synthesized peptide was conjugated to a carrier and used for immunization of rabbits or pigs. Serial boosts and bleeds were carried out and the titer measured by coimmunoprecipitation of cell-free translation products under native versus denatured conditions. Bleeds displaying a suitable titer under nondenaturing conditions were pooled and affinity purified on columns of the same peptide, in some cases after ammonium sulfate fractionation of total serum into an IgG-enriched fraction. After washing the peptide column, the affinity purified antibody was either eluted with 5 M magnesium acetate, followed by dialysis, or biotinylated, either on the column or after elution and dialysis into phosphate-buffered saline (PBS). Nonbiotinylated dialyzed antibody was used as the capture antibody, and the biotinylated antibody titer was determined to obtain the optimal signal-to-noise ratio, which varied from preparation to preparation. Rabbit antiserum was used for Western blotting with anti-rabbit secondary antibody coupled to HRP (Santa Cruz). NI controls for each antiserum demonstrated specificity in Western blots and immunoprecipitations. The specific peptides used for each plate screen (written from amino to carboxy terminal) were as follows: HIV-1, DRVHPVHAGPIAPGQMREPRGSDC; VEEV, CKTKKASKYDLEYADVPQSMR; HCV, CGVRATRKTSERSQPRGRRQPIPKAR; HNTV, GIQLGQRIIVLFMVAC; and RPXV, FLKYLLEHLIRSHYRVSKHITIVRYC.

### Plasmids and proviral constructs.

In this study, several variants of the HIV-1 NL4-3 proviral clone were utilized. For infection of PBMCs, we utilized the NL4-3 Infectious Molecular Clone (pNL4-3) from Malcolm Martin obtained through the NIH AIDS Reagent Program, Division of AIDS, NIAID, NIH (catalog number 114 [77]). For infection of MT-2, the pNL4-3 plasmid was modified to express *Renilla* luciferase in place of *nef* to make the HIV-1 pNL4-3 RLuc plasmid. For single-round MT-2 cell infections used to assay only HIV-1 early events, we introduced an *env* deletion (KpnI-BglII) into the RLuc plasmid to make pNL4-3 RLuc Δ*env*. We also used several variants of the HIV-1 LAI proviral clone. Chronically infected H9 and 293T cells were generated using HIV-1 pLAI *vpr*– *nef*– *puro*+, a proviral plasmid that contains a puromycin gene in place of *nef* for selection and two stop codons introduced into the NcoI site within *vpr* to inactivate Vpr (78). For single-round MT-2 cell infections used to evaluate viral late events, we used pLAI *pro*– Δ*env*, which we have described previously (30). The plasmid pcDNA NL4-3 Env used for pseudotyping was generated by cloning a Geneblock DNA fragment of codon-optimized NL4-3 *env* (Integrated DNA Technologies, Coralville, IA) into a pcDNA vector by Gibson assembly (79). For infection of SupT1.CCR5 cells, we utilized a molecular clone of a subtype A primary isolate, Q23-17 (80) that was modified to express a subtype A BG505 *env* (81, 82) and was obtained from Julie Overbaugh. Infection of G355-5 cells with FIV utilized the FIV-34TF10 ORFA+ proviral clone, described previously (31). The psPAX2 Gag-Pol helper plasmid was obtained from Didier Trono via Addgene (plasmid 12260; http://n2t.net/addgene:12260). To evaluate compound activity against FLUV, plasmids required for production of FLUV A/WSN/33 (83) were obtained from Andrew Pekosz.

### Cells and transfection.

The feline astrocyte cell line G355-5 was obtained from the American Type Culture Collection (ATCC; Manassas, VA; CRL-2033) and maintained in McCoy 5A (modified) medium supplemented with 10% fetal bovine serum (FBS; complete McCoy 5A). G355-5 cells were transfected with 1 to 6 μg of DNA in 6-cm dishes using X-treameGENE 9 (Roche, Basel, Switzerland). The 293T/17 cell line was obtained from ATCC (CRL-11268) and maintained in Dulbecco modified Eagle medium (DMEM) supplemented with 10% FBS and 1% penicillin-streptomycin (complete DMEM). 293T cells were transfected with 1 mg/ml polyethylenimine (PEI; Polysciences, Warrington, PA) at a PEI/DNA ratio of 3:1. The MT-2 cell line was obtained from Douglas Richman through the NIH AIDS Reagent Program, Division of AIDS, NIAID, NIH (catalog number 237 [84, 85]). SupT1.CCR5 cells, a SupT1 cell line stably expressing CCR5 HIV-1 coreceptor (86), were obtained from Julie Overbaugh. MT-2 and SupT1.CCR5 cells were maintained in RPMI supplemented with 10% FBS and 1% penicillin-streptomycin (complete RPMI). TZM-bl cells were obtained from John C. Kappes, and Xiaoyun Wu through the NIH AIDS Reagent Program, Division of AIDS, NIAID, NIH (catalog number 8129 [87–91]). The MDCK.2 cell line was obtained from ATCC (CRL-2986) and were maintained in minimal essential medium (MEM) supplemented with 10% FBS, 1% penicillin-streptomycin, 1% nonessential amino acid solution, and 1% sodium pyruvate (complete MEM).

### HIV-1 chronically infected cell lines.

The 293T-HIV and H9-HIV lines stably express the provirus LAI *vpr– nef– puro+*. These lines were generated by infecting either 293T/17 or H9 cells with NL4-3 *vpr– nef– puro+* at a multiplicity of infection (MOI) of 1, followed by selection with and maintenance in complete medium supplemented with 0.5 μg/ml puromycin.

### Antibodies for Western blotting and proximity ligation assay.

To detect HIV-1 Gag p24 in cell lysates and virus pellets by Western blotting (WB), we used αGag p24 mouse IgG_1_ monoclonal antibody isolated from the hybridoma 183-H12-5C obtained from Bruce Chesebro through the AIDS Reagent Program Division of AIDS, NIAID, NIH (catalog number 1513 [92]). Commercial antibodies were used to detect GAPDH (Abcam, Cambridge, MA; ab9485) and actin (Millipore-Sigma, St. Louis, MO; A2066) in cell lysates by WB. The isotype-specific secondary antibodies utilized for WB were obtained from Bethyl Laboratories (Montgomery, TX). For the proximity ligation assay (PLA), we utilized the αGag antibody described for the WB above, rabbit αbiotin IgG (Bethyl Laboratories, A150-109A), affinity purified rabbit αABCE1 IgG (which we have previously described [34]), mouse αbiotin IgG (Jackson Immunoresearch Labs, West Grove, PA; 200-002-211), mouse αDDX6 IgG (Millipore-Sigma, WH0001656M1), rabbit αDDX6 IgG (Bethyl Laboratories, A300-461A), rabbit αG3BP1 (Millipore-Sigma, PLA0231), mouse αTIAR (BD Biosciences, 610352), and rabbit αXRN1 (Millipore-Sigma, PLA0105). Rabbit IgG (Millipore-Sigma, I5006), and mouse IgG (Millipore-Sigma, I5381) were used for PLA NI controls.

### Virus infectivity measurement.

Infectivity was measured by titering stocks on TZM-bl cells. Cells seeded at 7,500 cells/well into 96-well polystyrene tissue culture plates were infected 24 h later with dilutions of culture supernatant or virus stock containing 20 μg/ml DEAE-dextran in a 50-μl volume for 4 h at 37°C. After infection, 150 μl of complete DMEM was added, and the plates were incubated at 37°C for 48 h. The cells were fixed with 1% formaldehyde–0.2% glutaraldehyde–PBS for 5 min and then washed with PBS. To determine the infectious units (IU), cells were incubated with 50 μl of X-Gal stain (4 mM potassium ferrocyanide, 4 mM potassium ferricyanide, 2 mM MgCl_2_, and 0.4 mg/ml 5-bromo-4-chloro-3-indolyl-β-d-galactopyranoside prepared in PBS) for 50 min at 37°C. After staining, the cells were washed several times with PBS, and blue cells (only wells with <100 cells) were counted to determine the IU/ml. To determine the relative infectivity, the cells were seeded, infected, fixed, and washed as described above, but instead of X-Gal stain, 100 μl of MUG stain (200 μg/ml 4-methylumbelliferone β-d-galactopyranoside prepared in DMEM) was added, followed by incubation at 37°C for 25 min. After incubation, the reaction was terminated with 100 μl of 1 M Na_2_CO_3_, and the well fluorescence was determined using 360 and 449 nm for excitation and emission settings, respectively (Synergy H1; BioTek Instruments, Inc., Winooski, VT). The relative fluorescence was used to compare infectivity between samples.

### PBMC activation.

Thawed, unstimulated PBMCs (BenTech, Seattle, WA) were pelleted by centrifugation 228 × *g* for 8 min in an Allegra centrifuge (Beckman, Brea, CA). Pellets were resuspended in activation medium containing RPMI supplemented with 21 ng/ml human interleukin-2 (IL-2; Peprotech, Rocky Hill, NJ; catalog no. 200-02), 1.5 μg/ml phytohemagglutinin (PHA; Remel, San Diego, CA; R30852801), 10% FBS, and 1% penicillin-streptomycin, at a density of 2 × 10^6^ cells/ml. After a 3-day incubation, the cells were collected and resuspended in RPMI with 20% FBS and 10% DMSO and then frozen as 50 × 10^6^ cells per vial.

### HIV-1 virus production.

To produce HIV-1 viral stocks 293T/17 cells were seeded at 10 × 10^6^ cells in a T-175 flask, transfected for 24 h, washed, and incubated in complete DMEM. Media from 48 and 72 h posttransfection collections were pooled and filtered using a 0.45-μm PES membrane, and 0.5-ml aliquots were stored at −80°C. To produce viral stocks of NL4-3, Q23.BG505, and LAI *vpr*– *nef*– *puro*+, 36 μg of proviral plasmids were used per T-175 flask. To produce viral stocks of NL4-3 RLuc Δ*env* pseudotyped with NL4-3 Env, 36 μg of the proviral plasmid and 12 μg of pcDNA NL4-3 Env were cotransfected. To produce viral stocks of LAI pro– Δ*env* pseudotyped with NL4-3 Env, 27 μg of proviral plasmid, 15 μg of pcDNA NL4-3 Env, and 18 μg of psPAX2 (Gag-Pol helper plasmid) were cotransfected.

### FLUV virus production.

FLUV A/WSN/33 viral stocks were prepared in MDCK.2 cells, as described previously (93).

### FIV virus production and RT assay.

G355-5 cells were transfected in six-well plates, and each well was transfected with 2 μg per well of FIV 34TF10 ORFA+ proviral plasmid. At 24 h posttransfection, the cells were washed, and the medium was replaced with 4 ml of complete McCoy 5A. At 144 h posttransfection, the medium was filtered using a 0.45-μm syringe filter, aliquoted, and stored at −80°C. The reverse transcriptase (RT) activity in media from FIV or mock-transfected cells was estimated using SG-PERT (94). A set of recombinant RT standards (EMD Millipore, Molsheim, France; catalog no. 382129) ranging from 5.12E3 to 5.12E9 nU/ml was used to estimate the nU/ml of RT activity in the FIV stock.

### Inhibition of HIV-1 replication. (i) MT-2 spreading infection assay.

Inhibition of HIV-1 viral replication was assayed in a spreading infection using MT-2 cells and NL4-3 RLuc reporter virus. For dose-response curves, compounds were initially diluted with DMSO to 100-fold the starting concentration in a 96-well plate and subjected to a series of 3-fold dilutions in DMSO for a total of eight or nine dilutions. If a single compound concentration was tested, the compound was diluted to 100-fold the desired concentration in DMSO. Compounds were then diluted 50-fold with infection media prepared by diluting NL4-3 RLuc virus stock to 400 IU/100 μl with complete RPMI. Then, 100 μl of 50-fold-diluted compound was transferred to 20,000 MT-2 cells that were preseeded in 96-well plates in 100 μl of complete RPMI for a final volume of 200 μl, followed by incubation at 37°C for 96 h. The final MOI in infected plates was 0.02, and the final DMSO concentration in all wells was 1%. All assays were run with three replicates. For each replicate, one well received DMSO only, and one well received medium only for normalization and background correction. To assay the inhibition of HIV-1 replication, 100 μl of medium was removed and discarded, and 10 μl of 15 μM EnduRen luciferase substrate was added to each well, followed by incubation for 1.5 h at 37°C. Plates were read on a luminescence plate reader (Synergy H1; BioTek Instruments, Inc.).

### (ii) SupT1.CCR5 spreading infection assay.

The SupT1.CCR5 assay was performed as described for the MT-2 spreading infection assay, with the following exceptions: 12.5 μl of Q23.BG505 virus stock was used per well, and inhibition of HIV-1 replication was determined by measuring the relative infectivity produced by infected cells using the MUG assay (see virus infectivity measurements above).

### (iii) PBMC spreading infection assay.

The PBMC assay was as described for the MT-2 spreading infection assay with the following exceptions. Frozen activated PBMCs were thawed and resuspended in complete PBMC medium containing RPMI supplemented with 21 ng/ml human IL-2 (Peprotech, catalog no. 200-02), 10% FBS, and 1% penicillin-streptomycin at 0.5 × 10^6^ cells/ml for 3 days in a 37°C tissue culture incubator. Cells were collected and adjusted to 2 × 10^6^ cells/ml in complete PBMC medium and bulk infected for 2 h with NL4-3 virus stock at an MOI of 0.008 at 37°C. Compound dilutions were prepared in complete PBMC medium as described above, but without virus, and 100 μl of diluted compound was transferred to 96-well U-bottom plates. Bulk-infected cells were pelleted and resuspended in complete PBMC medium at 1 × 10^6^ cells/ml, and 100 μl of cell suspension was transferred to each well at 100,000 cells per well with diluted compound. After 96 h, the medium was collected for relative infectivity measurements as for the SupT1.CCR5 spreading infection assay.

### FIV inhibition assay.

Inhibition of FIV spreading infection was assayed using G355-5 cells, seeded on the prior day at 8,000 cells/well in a 96-well plate. The preparation and addition of compounds were as in the MT-2 spreading infection assay with the following differences: for infection, 1,954 nU of RT activity of the FIV-34TF10 ORFA+ stock was used per well, infection assays proceeded for 144 h, and infectious virus production was measured using the SG-PERT assay described above.

### Virus pelleting.

For Fig. 3B, culture supernatants were collected at 48 h, and virus was pelleted by adding 75 μl of 17% PEG 6000–0.8 M NaCl–PBS to 75 μl of medium from the assay in a 96-well V-bottom polypropylene plate. PEG and media were incubated on ice for 2 h or overnight at 4°C. Virus was pelleted at 1,847 × *g* for 30 min at 4°C in the plate in an Allegra centrifuge (Beckman), and the pellet was resuspended in 50 μl of loading buffer with dithiothreitol for analysis by WB with αGag. For Fig. 4, culture supernatants were collected at 48 h postinfection and filtered through a 0.45-μm PES syringe filter to remove any remaining cells. Virus-like particles were centrifuged through a 30% (wt/vol) sucrose-PBS cushion at 130,000 × *g*, 30 min, and 4°C in an SW55Ti rotor (Beckman), and the virus pellet was harvested for analysis by WB with αGag.

### MT-2 acute infection assay.

MT-2 cells were diluted to 2 × 10^6^ cells/ml with complete RPMI and bulk infected at an MOI of 1 for 2 h at 37°C. Compound was diluted 100-fold to the final required concentration with DMSO. The compound dilutions and DMSO-only controls were then further diluted 50-fold with complete RPMI, and 1 ml of the dilutions was added per well in a 12-well plate. Bulk-infected cells were then washed and adjusted to 0.5 × 10^6^ cells/ml, and 1 ml of infected cells was added to each well containing diluted compound or DMSO. At 48 h, the medium was collected for virus pelleting as described above, and the cells were washed and lysed (see “Cell lysis” below).

### Cell lysis.

MT-2 cells were collected and washed three times by pelleting at 200 × *g* for 5 min at 4°C in an Allegra centrifuge (Beckman) and resuspending cells in ice-cold PBS. Cell pellets were lysed with 250 μl lysis buffer (20 mM HEPES [pH 7.9], 14 mM potassium acetate, 1 mM MgCl_2_, 0.3% NP-40), followed by shearing 20× with a 20-gauge needle. Cell lysate was then clarified 200 × *g* for 10 min at 4°C in an Allegra centrifuge (Beckman). The supernatant was transferred to a fresh tube and further clarified by centrifugation at 18,000 × *g* for 10 min at 4°C in a Microfuge (Beckman), and the supernatant was transferred to a fresh tube.

### Velocity sedimentation.

To prepare gradients, 10, 15, 40, 50, 60, 70, and 80% (wt/vol) sucrose solutions were prepared with water, and then each was diluted 1/10 with a 10× stock of the lysis buffer used to harvest cells (see “Cell lysis”). For each gradient, ∼100 μl of cell lysate (∼100 μg of total protein) was layered on a 5-ml step gradient containing equivalent layers of each diluted sucrose solution prepared and subjected to velocity sedimentation in a Beckman MLS-50 rotor 217,000 × *g* for 45 min at 4°C. Gradients were fractionated from top to bottom, and pelleted material was harvested for WB. Aliquots of fractions and pellet were analyzed by SDS-PAGE, followed by WB with αGag. Gag in pellet, which likely represents denatured Gag, was not included in the quantification of the velocity sedimentation gradients and represented <10% of total Gag signal in the gradient. The method for estimating the migration of particles with different S values in gradients has been described previously (28).

### Inhibition of HIV-1 early events.

For Fig. 3A, the assay described under MT-2 spreading infection assay was used with the following exceptions: MT-2 cells were infected with 0.5 μl/well of HIV-1 NL4-3 RLuc Δ*env* virus pseudotyped with NL4-3 Env, and luciferase was measured at 48 h instead of 96 h.

### Inhibition of HIV-1 late events.

For these experiments, we used H9-HIV, a chronically infected H9 T cell line. Compounds were diluted as described under MT-2 spreading infection above. To measure inhibition of infectious virus production (Fig. 3B, black graph), media were collected and relative infectivity of virus in the medium was measured using the MUG assay (see “Virus infectivity measurements” above). To measure inhibition of virus production (Fig. 3B, blue graph and blot), PEG pelleted virus (harvested as described in “Virus pelleting” above) was analyzed by WB with αGag.

### FLUV antiviral assay.

Inhibition of FLUV replication was assayed using infection of MDCK.2 cells. A day before the assay, MDCK.2 cells were seeded at 30,000 cells/well in complete MEM in a 96-well plate. The next day, seeded cells are washed with PBS and incubated for 1 h with 100 μl of FLUV A/WSN/33 stock diluted in VGM medium containing 42 mM HEPES, 0.125% bovine serum albumin, and 1 μg/ml TPCK (tolylsulfonyl phenylalanyl chloromethyl ketone)-trypsin (final MOI, 0.001). Cells were then washed with PBS and incubated in 90 μl of complete MEM. Single compound dilutions were prepared in complete MEM at 10× the desired final concentration, and 10 μl of DMSO or 10× compound was added to infected cells for final volume of 100 μl. At 24 h postinfection, the medium was aspirated, the cells were washed with PBS, and 100 μl of complete MEM was added to each well. After a 2 h of incubation, media were collected for 50% tissue culture infective dose (TCID_50_) determination.

### FLUV TCID_50_ assay.

FLUV TCID_50_ was assessed on MDCK.2 cells. MDCK.2 cells were seeded at 30,000 cells/well in complete MEM in a 96-well plate, and the next day the complete MEM was exchanged for 100 μl VGM media (as in the FLUV antiviral assay). Tenfold serial dilutions of virus-containing supernatant were prepared, and seven replicates of 11.1 μl of neat and diluted stocks were added to the MDCK cells (the final dilutions in the TCID_50_ plate ranged from 10^−1^ to 10^−6^). Plates were incubated at 37°C for 3 days, and the number of infected wells in the seven replicates for each dilution was determined by visual inspection. These data were used to calculate TCID_50_/ml by using the Reed and Muench method (95).

### Cytotoxicity assays.

For each viral inhibition assay, a cytotoxicity assay was performed in parallel on uninfected cells by adding 1/10 volume of alamarBlue (Thermo Fisher, Waltham, MA; DAL1199) to the plates, followed by thorough mixing. AlamarBlue-treated plates were incubated for 2 to 3 h at 37°C (the timing was optimized for the linear signal), mixed well, and read using a fluorescence plate reader with 560 nm and 590 nm as the excitation and emission settings, respectively (Synergy H1; BioTek Instruments, Inc.).

### Resistance assay.

Resistance selections were carried out in 12-well tissue culture plates. First, compound dilutions were prepared by adding 1.25 ml of complete RPMI to each well, followed by 25 μl of compound that was diluted to 100× the desired final concentration in DMSO. For the first passage, NL4-3 virus stock was then added to achieve an MOI of 0.01. For subsequent passages, 150 μl of the previous passage was added instead. Finally, 0.2 × 10^6^ MT-2 cells were added in 1.25 ml of complete RPMI. The cultures were incubated at 37°C until they reach the maximum viral cytopathic effect (CPE), as determined by visual inspection for the formation of syncytia (3 to 7 days). Selection was begun at 1×[EC_50_] and then increased 2-fold whenever maximum CPE was achieved in a time frame similar to that achieved by virus passaged in DMSO. At the end of each selection, the cells were pelleted from the media by centrifugation at 200 × *g* for 5 min in an Allegra centrifuge (Beckman), and the supernatant was transferred for storage at −80°C. PAV-206 and nelfinavir selections were carried out in parallel for 37 weeks.

### Sequencing virus from resistance selection.

The titer of the passaged medium was first determined on TZM-bl cells, and the IU/ml was determined by X-Gal (5-bromo-4-chloro-3-indolyl-β-d-galactopyranoside) staining (see “Virus infectivity measurement” above). The passaged virus was then used to infect MT-2 cells at an MOI of 1 in complete RPMI supplemented with 100 μg/ml DEAE dextran for 3 h at 37°C. The cells were pelleted at 200 × *g* for 5 min in an Allegra centrifuge (Beckman) and then resuspended in complete RPMI. At 15 h postinfection, the cells were washed three times with ice-cold PBS, and then viral cDNA from the cell pellet was harvested using a Spin miniprep kit (Qiagen, Venlo, Netherlands). Viral cDNA was amplified by PCR using Herculase II polymerase (Agilent, Santa Clara, CA), and amplified viral cDNA was gel purified using a spin gel purification kit (Qiagen). Two sets of primers were used to amplify viral cDNA in two overlapping fragments. The first half of the genome from 132 bp before the start of *gag* to midway through *vpr* was amplified with the forward primer 5′-AAGCGAAAGTAAAGCCAGAGG-3′ and the reverse primer 5′-AACGCCTATTCTGCTATGTCG-3′. The second half of the genome from *vpr* to 252 bp into *nef* was amplified with the forward primer 5′-CAGAGGACAGATGGAACAAGC-3′ and the reverse primer 5′-AGCTGCCTTGTAAGTCATTGG-3′. The long terminal repeat (LTRs) were not sequenced. Gel-purified PCR products were then subjected to Sanger sequencing. Gel chromatograms were visually inspected to identify a second chromatogram peak that was equal or larger in height than the peak corresponding to the reference sequence (defined as a ≥50% mutation; see Tables 1 and 2) or a single chromatogram peak that differs from the reference sequence (defined as a dominant mutation; see Tables 1 and 2).

### Proximity ligation assay.

For each treatment, one-half of a Grace Biolabs Culture Well silicone chamber (Millipore-Sigma; GBL103380) was attached to a cover glass (Carl Zeiss, Oberkochen, Germany; catalog no. 474030-9000-000) to create four chambers on the cover glass. Each chambered cover glass was placed into a well of a 6-well plate, and the chambers were coated with poly-l-lysine and allowed to dry overnight. The next day, ∼4 × 10^3^ 293T/17 or 293T-HIV cells were seeded into each chamber, followed by incubation at 37°C for 5 to 6 h. After the cells adhered, they were treated with compound or DMSO. At 16 h posttreatment, the cells were fixed for 15 min in 4% paraformaldehyde in PBS (pH 7.4), permeabilized in 0.5% (wt/vol) saponin in PBS (pH 7.4) for 10 min, and blocked in Duolink blocking solution (Millipore-Sigma) at 37°C for 30 min. The cells were incubated in primary antibodies using the following concentrations: for the Gag-biotin PLA, mouse αGag p24 was used at 0.2 μg/ml, and rabbit αbiotin was used at 4 μg/ml; for the biotin-ABCE1 PLA, rabbit αABCE1 was used at 0.3 μg/ml, and mouse αbiotin was used at 0.7 μg/ml; for the biotin-DDX6 PLA, rabbit αDDX6 was used at 0.5 μg/ml and mouse αbiotin was used at 0.3 μg/ml; for the ABCE1-DDX6 PLA, rabbit αABCE1 was used at 0.3 μg/ml, and mouse αDDX6 was used at 0.3 μg/ml; for the biotin-G3BP1 PLA, rabbit αG3BP1 was used at 0.025 μg/ml, and mouse αbiotin was used at 0.2 μg/ml; for the TIAR-G3BP1 PLA, rabbit αG3BP1 was used at 0.025 μg/ml, and mouse αTIAR was used at 0.025 μg/ml; for the biotin-XRN1 PLA, rabbit α XRN1 was used at 0.5 μg/ml, and mouse αbiotin was used at 0.2 μg/ml; and for the DDX6-XRN1 PLA, rabbit αXRN1 was used at 0.5 μg/ml, and mouse αDDX6 was used at 0.5 μg/ml. For the NI control PLA experiments, NI control antibodies from the relevant species were used at the same concentration as the primary antibody they replaced. Primary incubation was followed by incubation with Duolink reagents (Millipore-Sigma): oligonucleotide-linked secondary antibody, ligation mix, and red amplification/detection mix, with washes in between, in accordance with the Duolink protocol. For concurrent IF analysis following the final 1× PLA buffer B washes, cells were incubated for 30 min at room temperature with 1:1,000 Alexa Fluor 488-anti-mouse secondary antibody. Cover glasses were mounted using Duolink *in situ* mounting medium with DAPI (4′,6′-diamidino-2-phenylindole; Millipore-Sigma), sealed to the glass slides with clear nail polish, allowed to dry for 24 h at room temperature, and stored at −20°C. Imaging was performed with a Zeiss Axio Observer.Z1/7 deconvolution microscope using Zeiss Plan-Apochromat 63×/aperture 1.4 objective with oil immersion, with Zen 2.6 (blue edition) software. For quantification, 5 to 10 fields, each containing at least three IF-positive cells, were chosen at random and imaged using identical exposure times for each channel (100 to 150 ms for the PLA channel in red; 0.5 to 1 s for the IF channel in green, depending on the IF primary antibody; and 20 ms for the DAPI channel in blue). Images were captured as a Z-stack of 80 to 90 0.24-μm slices that included the entire cell body and 5 μm above and below the cell (∼20 μm total). To determine the number of PLA spots per cell, image stacks were processed as follows in Fiji (96). First, an in-focus slice from the IF channel was selected using the Find Focused Slices plugin within ImageJ (Q. Tseng, 2020 [https://sites.google.com/site/qingzongtseng/find-focus]), and thresholding was used to generate regions of interest (ROI) that contained only cells. The ROIs identified in the IF channel were applied to a maximum intensity *z* projection of the PLA channel, and PLA spots within the ROI were counted using the find maxima function in Fiji, with noise threshold optimized to identify only PLA spots. Nuclei from each field were counted manually to determine the number of cells. Total PLA spots counted were divided by total nuclei to obtain the average number of PLA spots per cell, and these results were plotted with error bars representing the standard errors of the mean (SEM), with *n* being the total number of cells counted. The numbers of fields and cells analyzed for each PLA were as follows: mouse Gag-rabbit biotin PLA (Fig. 6), 20 fields, containing a total of 186 to 316 cells; rabbit ABCE1-mouse biotin PLA (Fig. 10), 10 fields, containing a total of 104 to 155 cells; rabbit DDX6-mouse biotin PLA (Fig. 11), 5 fields, containing a total of 39 to 73 cells; mouse DDX6-rabbit ABCE1 PLA (Fig. 9), 10 fields, containing a total of 121 cells; mouse Gag-rabbit biotin PLA (Fig. 7), 20 fields, containing a total of 213 cells; rabbit biotin-mouse NI PLA (Fig. 7), 10 fields, containing a total of 118 cells; rabbit NI-mouse Gag PLA (Fig. 7), 10 fields, containing a total of 131 cells; rabbit ABCE1-mouse biotin PLA (Fig. 7), 5 fields, containing a total of 66 cells; mouse biotin-rabbit NI PLA (Fig. 7), 5 fields, containing a total of 59 cells; mouse NI-rabbit ABCE1 (Fig. 7), 5 fields, containing a total of 77 cells; rabbit DDX6-mouse biotin PLA (Fig. 7), 5 fields, containing 50 cells; rabbit NI-mouse biotin PLA (Fig. 7), 5 fields, containing 51 cells; rabbit DDX6-mouse NI (Fig. 7), 5 fields, containing 43 cells; mouse biotin-rabbit G3BP1 PLA (Fig. 12), 5 fields, containing 63 to 83 cells; mouse TIAR-rabbit G3BP1 PLA (Fig. 12), 5 fields, containing 63 to 75 cells; mouse TIAR-rabbit NI (Fig. 13), 5 fields, containing 45 cells; mouse NI-rabbit G3BP1 PLA (Fig. 13), 5 fields, containing 60 cells; mouse TIAR-rabbit G3BP1 PLA (Fig. 13), 5 fields, containing 62 cells; mouse DDX6-rabbit NI (Fig. 13), 5 fields, containing 46 cells; mouse NI-rabbit XRN1 PLA (Fig. 13), 5 fields, containing 54 cells; mouse DDX6-rabbit XRN1 PLA (Fig. 13), 5 fields, containing 73 fields; mouse DDX6-rabbit XRN1 PLA (Fig. 14), 5 fields, containing 57–73 cells; and mouse biotin-rabbit XRN1 PLA (Fig. 14), 5 fields, containing 44 to 54 cells. For the presentation of images in figures, a central slice of the image stack was chosen, and a region of the field of 1,875 pixels × 938 pixels (Fig. 6, 7, 9, 10, 11, and 13) or 990 pixels × 938 pixels (Fig. 12 and 14) was selected and adjusted as follows. For all channels, background was removed by setting the intensity minimum above the background signal. For the PLA channel, the gain on PLA spots was increased for visualization by setting the channel intensity maximum to approximately one-third the intensity of the most intense pixel in the selected field. For the IF channel, IF signal was reduced to improve PLA spot visualization by setting the channel intensity maximum to approximately twice the intensity of the most intense pixel in the selected field. A grayscale image of the PLA channel only and an RGB image of the merged channels (PLA, red; IF, green; and DAPI, blue) were generated and imported into Inkscape, an open-source vector graphics editor, to create the final figure layout without further adjustment.

### Statistical analysis.

EC_50_ and CC_50_ values were determined in Excel using four-parameter logistic regression analysis of dose-response data generated from the viral inhibition or cytotoxicity assays, respectively. Solid lines on dose-response plots represent the fitted logistic regression. Average EC_50_ and CC_50_ values were calculated using the geometric mean and average values were reported with the geometric standard deviation (GSD). The statistical difference between pairs of treatment groups in the PLA experiments was analyzed in Excel using a two-tailed unpaired Student *t* test assuming unequal variance.

### Synthesis of compounds.

See Supplemental Methods S1 in the supplemental material.

## Supplementary Material

Supplemental file 1
