# Supplementary material for: Identification of an Antiretroviral Small Molecule That Appears To Be a Host-Targeting Inhibitor of HIV-1 Assembly
Source: J Virol. 2021 Jan 13;95(3):e00883-20. doi: 10.1128/JVI.00883-20 (PMC7925099; doi:10.1128/JVI.00883-20)
Supplement: Supplemental file 1 [file JVI.00883-20-s0001.pdf]

Synthesis of Tetrahydroisoquinolines

General Procedure for the Synthesis of Substituted Phenethyl amines[a]

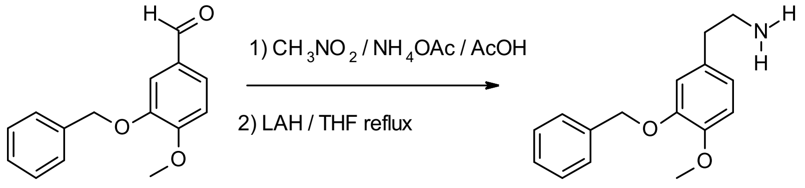

1 2

[0001] A solution of the benzaldehyde **1** (0.1 mol), nitromethane (50 mL, 0.93 mmol) and ammonium acetate (0.26 mol) in acetic acid (200 mL) was refluxed for 1 h. Upon cooling, the product crystallized out of solution. The crystals were filtered off and washed with a small amount of ether to give a bright yellow nitrostyrene.

[0002] To a stirred solution of lithium aluminum hydride (0.21 mol) in tetrahydrofuran (270 mL) was added dropwise a solution of the above nitrostyrene (0.11 mol) in tetrahydrofuran (200 mL). Upon completion, the reaction mixture was refluxed with stirring for 16 h, cooled to room temperature, then the excess hydride was decomposed by the addition of aq. sat. Na_2SO_4. The mixture was filtered and the filtrate rotary evaporated to an amber–brown oil (the title compound, **2)** which was used without further purification.

General Procedure for the Synthesis of Substituted Cinammic acids[b]

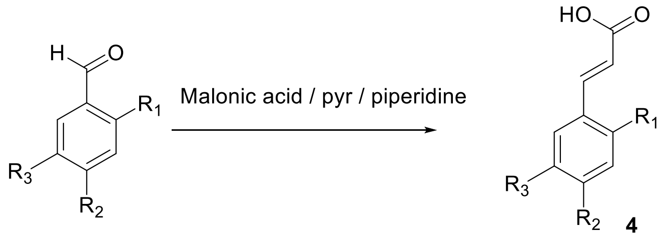

3

[0003] A mixture of the benzaldehyde **3** (30 mmol), malonic acid (60 mmol), pyridine (20 mL) and piperidine (5 mmol) was stirred at 80°C for 1 h followed by refluxing at 110-115°C for an additional 3 h. The cooled reaction mixture was poured into water (250 mL) and acidified with conc. HCl. The resulting precipitate was filtered off and washed several times with water. It was redissolved in 2M NaOH, diluted with water, then acidified with conc. HCl. The solid precipitate was filtered, washed several times with water, then dried under high vacuum over P_2O_5 to afford the title compound **4**.

General Procedure for the Synthesis of Substituted Cinnamides[c]

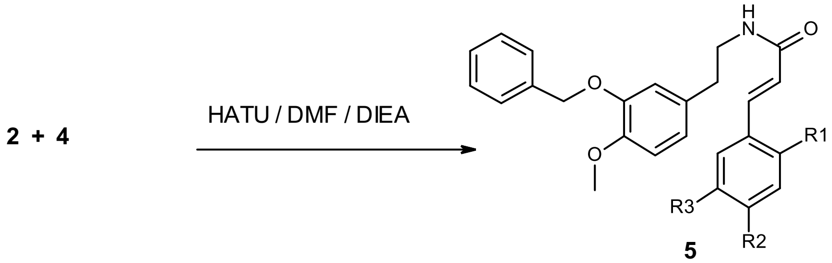

5

[0004] To a stirred mixture of the substituted phenethylamine **2** (0.48 mmol), substituted cinnamic acid **4** (0.72 mmol), DIEA [420 µL (2.4 mmol)] & 10 mL of DMF was added HATU (1 mmol). The reaction was stirred at RT for 1 h and then diluted with 20 mL of EtOAc and washed twice with sat NaCl. The EtOAc layer was dried (Na_2SO_4) and the solvent removed, yielding the title compound **5**.

General Procedure for the Cyclization and Reduction of the Substituted Cinnamides: Synthesis of Substituted Tetrahydroisoquinolines[d]

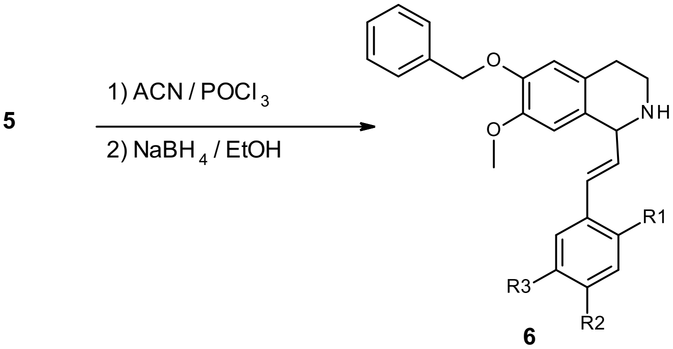

6

[0005] To the substituted cinnamide **5** (0.563 mmol) in ACN (13 mL) was added, under reflux, $POCl_3$ (3.9 mmol). The reaction was stirred at reflux for 30 min and then rotary evaporated to dryness. The residue was taken up into 10 mL of chloroform and was then treated with 20 mL of 2N KOH and 50 mL of Et_2O. This mixture was rapidly stirred for 30 min at room temperature. Then the upper organic layer removed, washed with water, dried (Na_2SO_4), and the solvent removed. The resulting dark oil (substituted dihydroisoquinoline) was then dissolved into 8 mL of dry EtOH then treated with $NaBH_4$ (0.395 mmol). The excess reagent was destroyed by dropwise addition of 2M HCl, basified with 2M KOH and evaporated to dryness to remove EtOH. The residue obtained was partitioned between water and $CHCl_3$, the organic layer was washed with brine, dried (K_2CO_3), filtered, concentrated and purified by flash chromatography

using 0.4M NH_3 in MeOH/ $CHCl_3$ gradient (and/or purification via RP C18 Prep HPLC using 0.1% TFA acetonitrile /water) to afford the title compound **6**.

All compounds were confirmed by Mass Spectrometry:

	R_1	R_2	R_3	M+H (exact mass + H)
PAV117	CH_3	O-CH_3	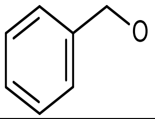	522
PAV218	CH_3	O-CH_3	H	416
PAV220	H	O-CH_3	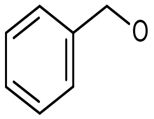	508
PAV494	H	H	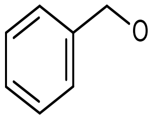	478
PAV493	H	O-CH_3	H	402
PAV245	CH_3	H	H	386
PAV492	H	H	H	372
PAV206	CH_3	O-CH_3	O-CH_3	446

[a] Cava, M.P.; Buck, K.T. *Tetrahedron* 1969, 25, 2795-2805

[b] Nag, Ahindra et al *Journal of Molecular Catalysis B: Enzymatic* **2012**, 82, 92-95; Heo, J.N., et al., *Bull. Korean Chem. Soc.*, 32 (12), 4431, **2011**; Srikrishna, A. et al, *Synthetic Communications*, 37(6), 965-976; **2007**.

[c] Patent reference: WO2010001169, Astrazeneca.

[d] Herbert, Richard B. et al *Tetrahedron* **1990**, 46, 7119-7138.

Synthesis of N-[2-[2-[3-[[2-[2-[4-[5-[(E)-2-(6-benzyloxy-7-methoxy-1,2,3,4-tetrahydroisoquinolin-1-yl)vinyl]-2-methoxy-4-methyl-phenoxy]butanoylamino]ethylamino]-1-[(3-methyldiazirin-3-yl)methyl]-2-oxo-ethyl]amino]-3-oxo-propoxy]ethoxy]ethyl]-5-[(4S)-2-oxo-1,3,3a,4,6,6a-hexahydrothieno[3,4-d]imidazol-4-yl]pentanamide (PAV-818)

Synthesis of 5-(4-bromobutoxy)-4-methoxy-2-methyl-benzaldehyde:

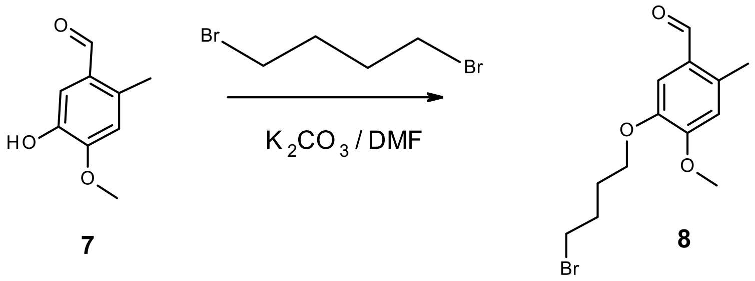

7 **8**

To a solution of aldehyde **7** **[**2.7 g (0.016 mol)] in 30 mL of dry DMF was added K_2CO_3 [6.9 g (0.05 mol)]. While stirring at room temperature under argon atmosphere was added dropwise 1,4-dibromobutane [10 g (0.048 mol)]. After stirring overnight at room temperature, the reaction mixture was diluted with 100 mL of EtOAc then washed twice with water. The organic layer was dried (Mg_2SO_4), filtered, and rotary evaporated to dryness to afford 4.1 g of the desired bromide **8**.

Synthesis of 5-(3-azidopropoxy)-4-methoxy-2-methyl-benzaldehyde:

8

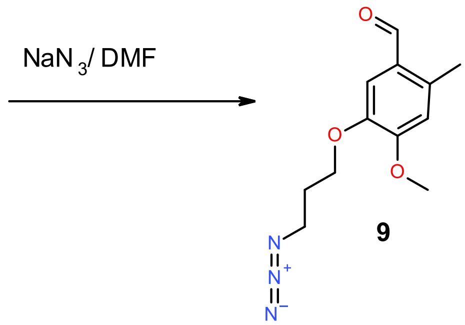

9

To a solution of aldehyde **8** **[**4.1 g (0.014 mol)] in 30 mL of dry DMF was added NaN_3 [1.3 g (0.02 mol)]. After stirring at 70°C under argon atmosphere for 5 h, the reaction mixture was cooled then diluted with 100 mL of EtOAc, followed by washing twice with water. The

organic layer was dried (Mg_2SO_4), filtered, and rotary evaporated to dryness to afford 3.3 g (94%) of the desired azide **9**.

<u>Synthesis of tert-butyl 1-[(E)-2-[5-(4-azidobutoxy)-4-methoxy-2-methyl-phenyl]vinyl]-6-benzyloxy-7-methoxy-3,4-dihydro-1H-isoquinoline-2-carboxylate:</u>

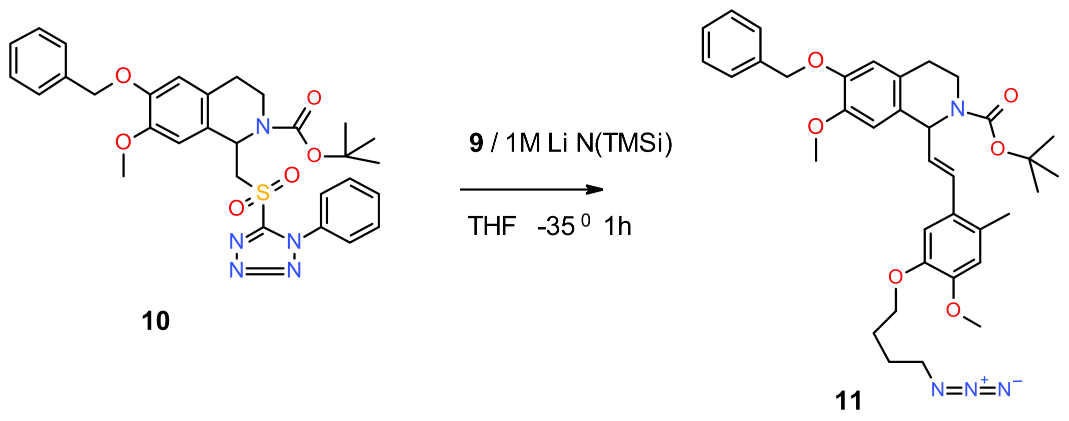

To a solution of azide **9** [315 mg (1.2 mmol)] in 8 mL of dry THF was added the tetrahydroisoquinoline **10**[e] [236 mg (0.4 mmol)]. The mixture was then cooled and stirred at −35°C under argon atmosphere. Next 1M LiHMDS in THF [1.2 mL (1.2mmol)] was slowly added dropwise and upon complete addition, the reaction mixture was stirred at −35°C for 1 h. The reaction mixture was allowed to come to room temperature then quenched with 15 mL of sat NH_4Cl followed by extraction with 20 mL of EtOAc. The organic layer was dried (Mg_2SO_4), filtered, and rotary evaporated to dryness to afford the crude azide **11**. After flash column chromatography using a gradient of EtOAc / hexane, pure **11** (135 mg) was obtained in 54% yield.

Synthesis of tert-butyl 1-[(E)-2-[5-(4-aminobutoxy)-4-methoxy-2-methyl-phenyl]vinyl]-6-benzyloxy-7-methoxy-3,4-dihydro-1H-isoquinoline-2-carboxylate:

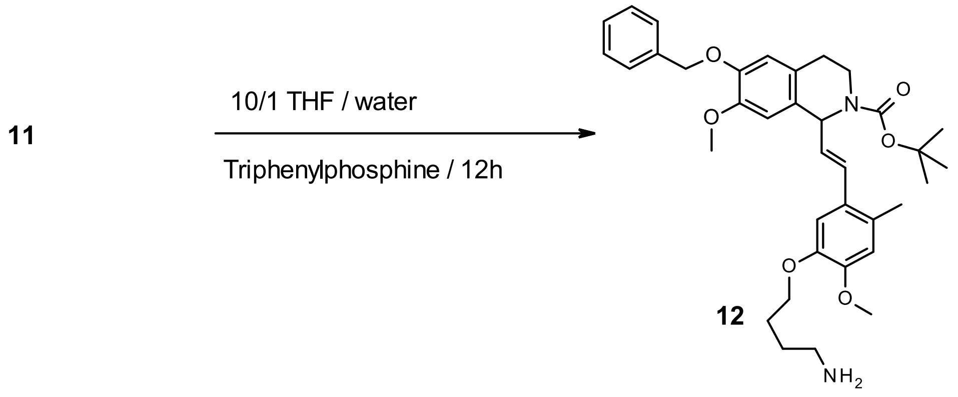

To a solution of azide **11** [135 mg (0.215 mmol)] in 5 mL of a 10/1 THF / water was added triphenylphosphine [64 mg (0.25 mmol)]. The reaction mixture was stirred at room temperature for 12 h. The reaction mixture was then diluted with 10 mL of water and extracted with 20 mL of EtOAc. The organic layer was dried (Mg_2SO_4), filtered, and rotary evaporated to dryness to afford the crude amine **12**. After flash column chromatography using a gradient of 0.4M NH_3 in MeOH/ $CHCl_3$, pure **12** (90 mg) was obtained in 70% yield.

Synthesis of 3-(3-methyldiazirin-3-yl)-2-[3-[2-[2-[5-[(4S)-2-oxo-1,3,3a,4,6,6a-hexahydrothieno[3,4-d]imidazol-4-yl]pentanoylamino]ethoxy]ethoxy]propanoylamino]propanoic acid:

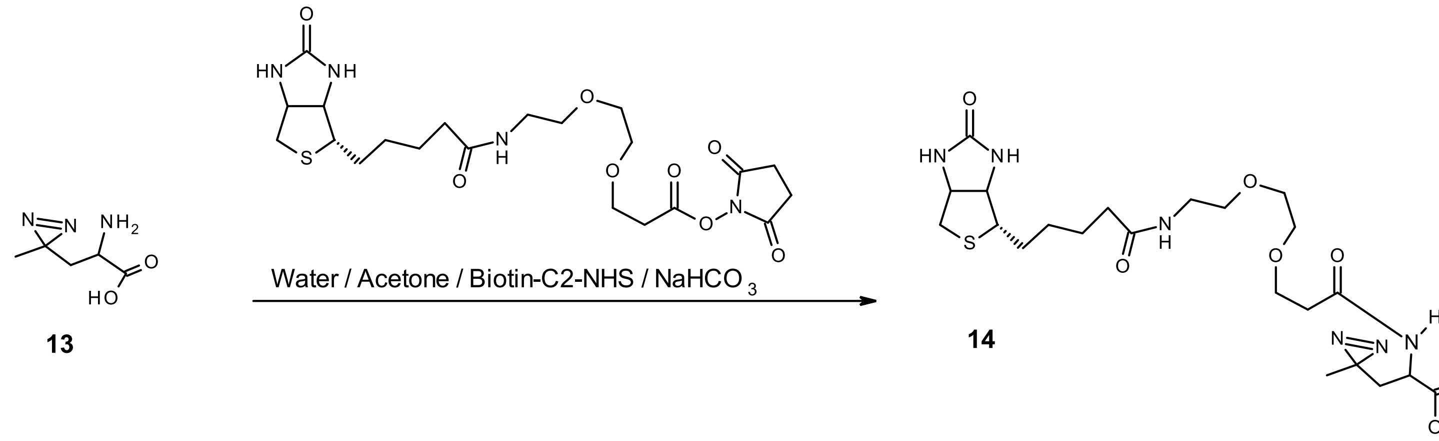

To a mixture of diazirine **13**[f] **[**25 mg (0.17 mmol)], 1 mL acetone, 1 mL water and $NaHCO_3$ [43 mg (0.17 mmol)] was added, with vigorous stirring, Biotin-C2-NHS[f] [85 mg (0.17 mmol)]. The reaction mixture was stirred at room temperature for 12 h. The reaction mixture was then diluted with 5 mL of water and extracted with 15 mL of EtOAc. The organic layer was dried (Mg_2SO_4), filtered, and rotary evaporated to dryness to afford the crude acid **14** (40 mg). This was used as was in the next reaction.

<u>Synthesis of N-[2-[2-[3-[[2-[4-[5-[(E)-2-(6-benzyloxy-7-methoxy-1,2,3,4-tetrahydroisoquinolin-1-yl)vinyl]-2-methoxy-4-methyl-phenoxy]butylamino]-1-[(3-methyldiazirin-3-yl)methyl]-2-oxo-ethyl]amino]-3-oxo-propoxy]ethoxy]ethyl]-5-[(4S)-2-oxo-1,3,3a,4,6,6a-hexahydrothieno[3,4-d]imidazol-4-yl]pentanamide (PAV-818):</u>

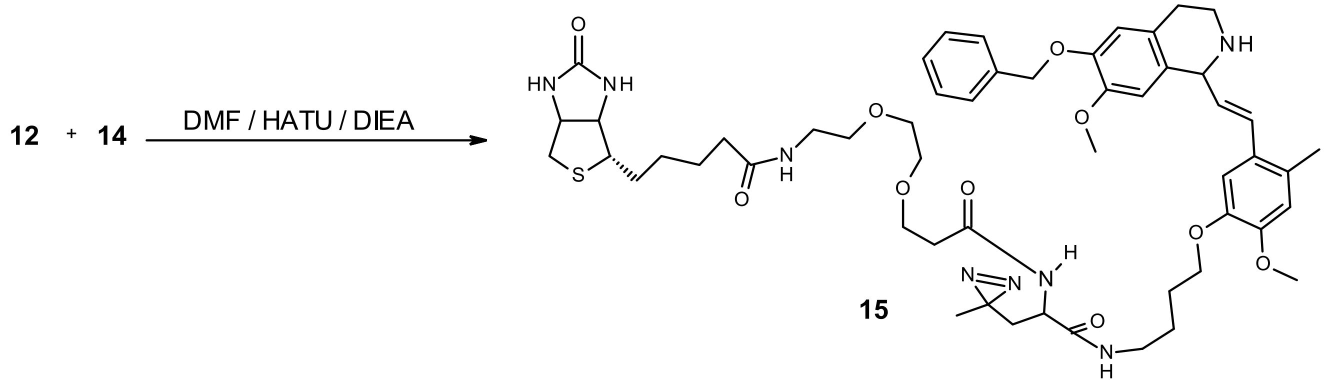

To a mixture of acid **14 [**40 mg (0.076 mmol)] and amine **12** [(45 mg (0.075 mmol)] was added 1 mL of dry DMF and DIEA [35 µl (0.2 mmol)]. Next HATU [38 mg (0.1 mmol)] was added in one portion and the resulting mixture was stirred at room temperature for 0.5 h. The reaction mixture then diluted with 5 mL of EtOAc, followed by washing twice with water. The organic layer was dried (Mg_2SO_4), filtered, and rotary evaporated to dryness. The residue was taken up into 1 mL of acetonitrile / water (90 /10) then purified on a RP C18 Prep HPLC using 0.1% TFA acetonitrile /water to afford 17 mg (22% overall) of the desired material **15**.
M+H=1013

Synthesis of 2-Amino-3-cyano-N-(4-phenoxybutyl)propanamide;N-[2-[2-[3-[[1-(cyanomethyl)-2-oxo-2-(4-phenoxybutylamino)ethyl]amino]-3-oxo-propoxy]ethoxy]ethyl]-5-[(4S)-2-oxo-1,3,3a,4,6,6a-hexahydrothieno[3,4-d]imidazol-4-yl]pentanamide (PAV-543)

Synthesis of tert-Butyl N-[1-(cyanomethyl)-2-oxo-2-(4-phenoxybutylamino)ethyl]carbamate:

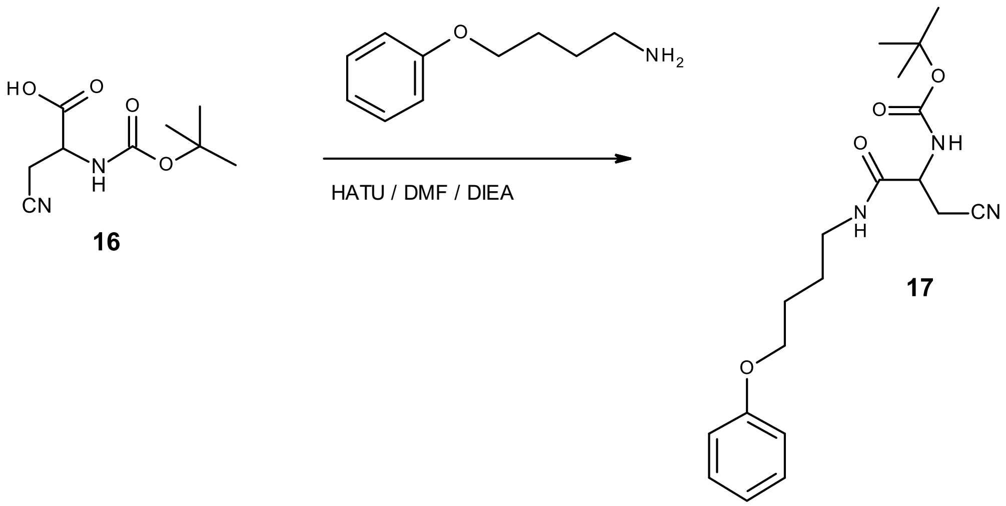

To a mixture of acid **15**[f] [214 mg (1 mmol)] and 4-phenoxybutan-1-amine[f] [(165 mg (1 mmol)] was added 5 mL of dry DMF and DIEA [520 μl (3 mmol)]. Next HATU [460mg (1.2 mmol)] was added in one portion and the resulting mixture was stirred at room temperature for 0.5 h. The reaction mixture then diluted with 5 mL of EtOAc, followed by washing twice with water. The organic layer was dried (Mg_2SO_4), filtered, and rotary evaporated to dryness. The crude compound **17** was used as was.

<u>Synthesis of tert-Butyl N-[1-(cyanomethyl)-2-oxo-2-(4-phenoxybutylamino)ethyl]carbamate:</u>

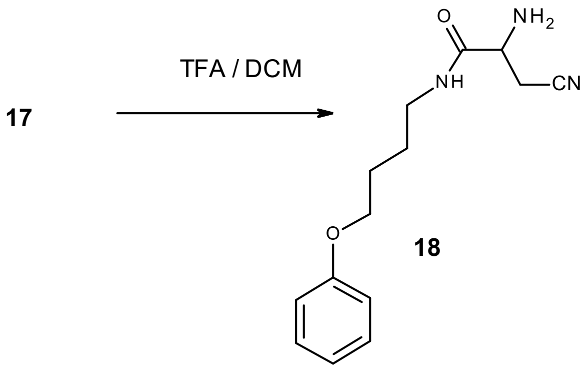

The amide **17** was treated with a mix of TFA / DCM (3/5) [5mL] for 5 min and again rotary evaporated to dryness. This crude amine **18** was taken on to the next step as a TFA salt.

Synthesis of 2-Amino-3-cyano-N-(4-phenoxybutyl)propanamide;N-[2-[2-[3-[[1-(cyanomethyl)-2-oxo-2-(4-phenoxybutylamino)ethyl]amino]-3-oxo-propoxy]ethoxy]ethyl]-5-[(4S)-2-oxo-1,3,3a,4,6,6a-hexahydrothieno[3,4-d]imidazol-4-yl]pentanamide (PAV-543):

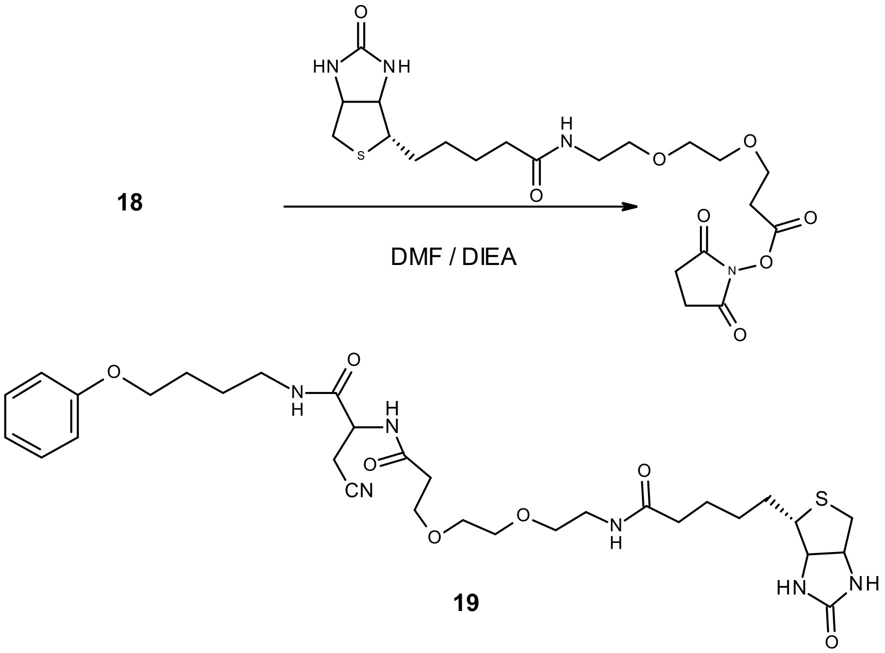

To a mixture of the amine salt **18**, 4 mL DMF and DIEA [520µL (3 mmol)] was added, with vigorous stirring, Biotin-Peg2-NHS[f] [500mg (1 mmol)]. The reaction mixture was stirred at room temperature for 12 h. The reaction mixture was then diluted with 5 mL of water and extracted with 15 mL of EtOAc. The organic layer was dried (Mg_2SO_4), filtered, and rotary evaporated to dryness to afford the crude amide **19.** The crude material was taken up into 3 mL of acetonitrile / water (90 /10) and then purified on a RP C18 Prep HPLC using 0.1% TFA acetonitrile /water to afford 97 mg (15% overall) of the desired material **19**. M+H=647

[e] Reddy, R. et al *Journal of Organic Chemistry* 2012, 77, 11101-11108

[f] commercially available
